# Phenotypic Heterogeneity in Genetic and Acquired Pediatric Cerebellar Disorders

**DOI:** 10.1002/mds.30210

**Published:** 2025-05-06

**Authors:** Katariina Granath, Sanna Huhtaniska, Juulia Ellonen, Tytti Pokka, Salla M. Kangas, Jukka Moilanen, Heli Helander, Hanna Kallankari, Jonna Komulainen‐Ebrahim, Päivi Vieira, Elisa Rahikkala, Renzo Guerrini, Minna Honkila, Terhi S. Ruuska, Reetta Hinttala, Maria Suo‐Palosaari, Jussi‐Pekka Tolonen, Johanna Uusimaa

**Affiliations:** ^1^ Department of Paediatrics and Adolescent Medicine, Division of Paediatric Neurology Oulu University Hospital Oulu Finland; ^2^ Research Unit of Clinical Medicine, University of Oulu and Medical Research Center Oulu, Oulu University Hospital and University of Oulu Oulu Finland; ^3^ Department of Diagnostic Radiology, Physics and Technology, Research Unit of Health Sciences and Technology, and Medical Research Center Oulu Oulu University Hospital and University of Oulu Oulu Finland; ^4^ Research Service Unit, Research Unit of Clinical Medicine Oulu University Hospital Oulu Finland; ^5^ Department of Clinical Genetics Oulu University Hospital, Oulu University Hospital Oulu Finland; ^6^ Neuroscience Department Meyer Children's Hospital IRCCS and University of Florence Florence Italy; ^7^ Department of Paediatrics and Adolescent Medicine Oulu University Hospital Oulu Finland; ^8^ Biocenter Oulu, University of Oulu Oulu Finland

**Keywords:** ataxia, cerebellum, neurogenetics, neuroimaging, pediatric

## Abstract

**Background:**

The genetic landscape of pediatric cerebellar disorders (PCDs) in Finland is undefined.

**Objectives:**

The objective was to define epidemiological, clinical, neuroradiological, and genetic characteristics of PCDs in Northern Finland.

**Methods:**

A longitudinal population‐based cohort study of children with a movement disorder or a cerebellar malformation (diagnosis ≤16 years; study period 1970–2022) was performed in the tertiary catchment area of the Oulu University Hospital, Finland. The genotype‐to‐phenotype associations were compared with 1007 published cases with matching monogenic etiologies.

**Results:**

A total of 107 patients were included (cumulative incidence 21.9 per 100,000 live births). A defined genetic or non‐genetic etiology was identified for 59 patients. These etiologies were monogenic (66%), chromosomal (12%), or non‐genetic (22%). Ataxia was the most common movement disorder. Friedreich's ataxia was uncommon, whereas ataxias belonging to the Finnish Disease Heritage were overrepresented. Forty‐eight cases remained undefined. The diagnostic yield (ie, pathogenic or likely pathogenic variants) of next‐generation sequencing (NGS) in ataxia was 65%. Common features were ataxia, developmental delay, seizures, hypotonia, and abnormality in brain MRI, whereas hearing loss, sensory neuropathy, and microcephalia were associated with fewer etiologies.

**Conclusions:**

PCDs are a heterogeneous disease group with a high proportion of genetic etiologies. Age of onset and certain clinical findings may help distinguish between different disease entities. The diagnostic yield of NGS has increased over time. Our dataset will support clinicians to recognize PCDs, their co‐morbidities, and genetic etiologies. Further data on epidemiology, shared disease mechanisms, and the natural history of PCDs will be critical for the development of treatment approaches. © 2025 The Author(s). *Movement Disorders* published by Wiley Periodicals LLC on behalf of International Parkinson and Movement Disorder Society.

Pediatric cerebellar disorders (PCDs) are childhood‐onset neurological diseases that affect the cerebellum. Patients may display various combinations of movement disorders, cognitive deficits, communication disorders, extra‐cerebellar deficits in the central or peripheral nervous systems, and multiorgan involvement. In clinical practice, categorizing PCDs as transient developmental disorders; paroxysmal movement disorders; disorders with secondary, non‐inherited causes; hereditary; or metabolic disorders can provide a helpful framework for differential diagnosis.^1^, ^2^ Neuroimaging is a central biomarker to facilitate the interpretation of next‐generation sequencing (NGS) data.^1^, ^3^

In 2013, the estimated prevalence in Europe of cerebellar ataxias with childhood‐onset (aged 0–19 years) was 26/100,000, with 54% of cases being genetic ataxias.^4^ Among cases of genetic ataxia across the human lifespan, most (59%–62.5%) are diagnosed as autosomal recessive ataxias.^5^, ^6^ However, as many as 60% of patients with childhood‐onset ataxia lack a specific molecular diagnosis.^7^, ^8^ This diagnostic yield, as established by Németh et al (2013) and Ignatius et al (2020), has not increased in nearly a decade,^7^, ^8^ prompting us to study PCDs and their etiologies.

Finland has a unique genetic landscape with enrichment of genetic variants that are rare in other European populations: The Finnish Disease Heritage (FDH)^9^ enables the identification of novel genetic etiologies in neurodevelopmental disorders. As the epidemiology of PCDs in Finland is yet to be described, we designed our cohort study (Pediatric Cerebellar Ataxias—Genetic Landscape of Northern Finland [PEDIATAX]) to evaluate the epidemiology, etiologies, and clinical and neuroradiological characteristics of PCDs in Northern Finland. Ultimately, our dataset—which also includes a comprehensive review of 1007 clinical cases from 535 published case reports—provides a practical tool to support clinical diagnostic approaches in PCDs, a basis for precision medicine and possible shared disease mechanisms, and a focus for the development of collective treatment options for rare ataxias.

## Patients and Methods

### Study Setting

**FIG. 1:**
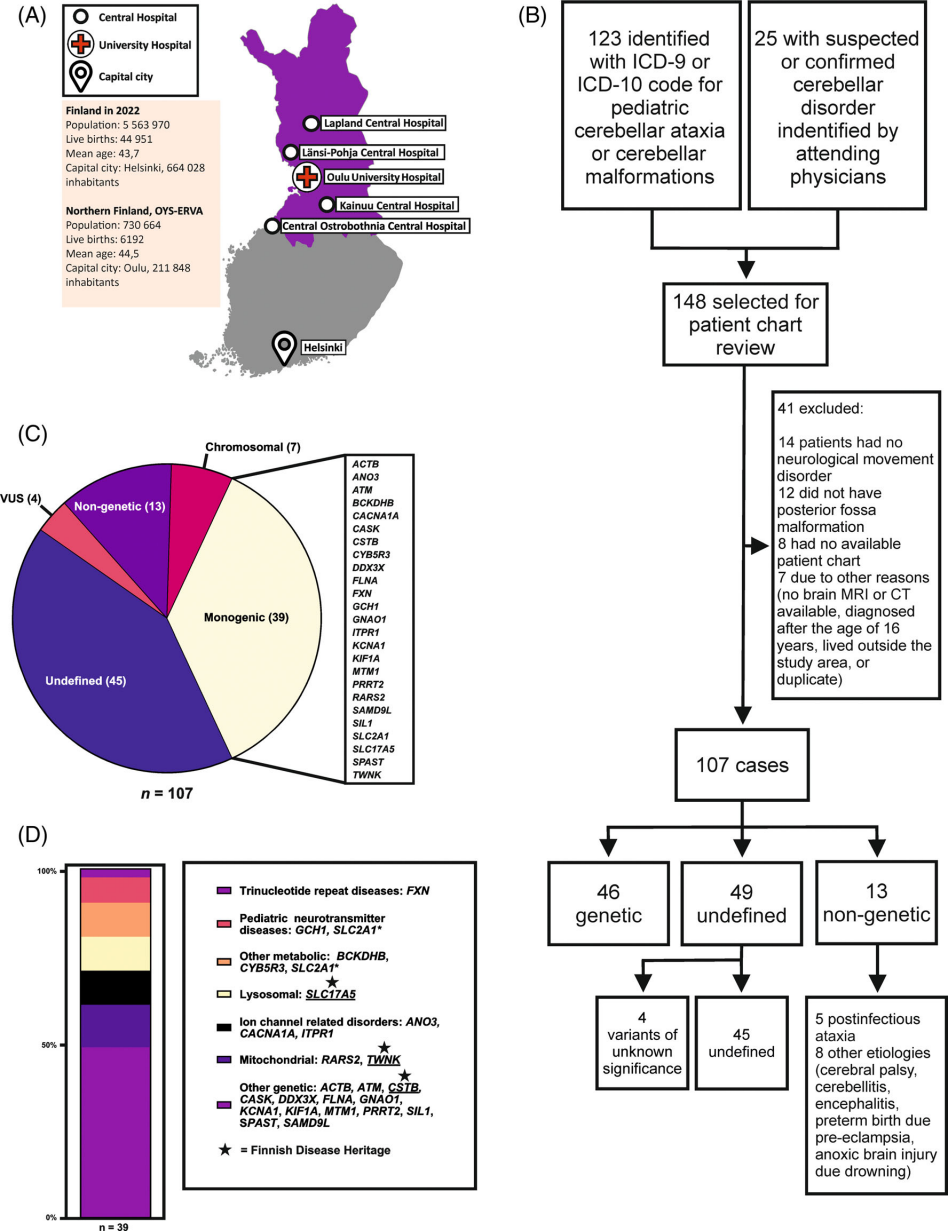
Location and composition of the PEDIATAX cohort. (**A**) The Oulu University Hospital is the only tertiary care center specialized in child neurology in Northern Finland (dark violet). (**B**) Establishment of the PEDIATAX cohort. (**C**) Etiologies for PCDs and number of patients in each group. One patient has been included in two categories. (**D**) Proportions of PCDs, adapted from O'Malley et al. SLC2A1* = The GLUT1 deficiency syndrome caused by variants in the *SLC2A1* gene can be classified as both a metabolic disease and a pediatric neurotransmitter disease. CT, computed tomography; ICD, *International Classification of Diseases*; MRI, magnetic resonance imaging; PCD, pediatric cerebellar disorder; VUS, variant of unknown significance. [Color figure can be viewed at wileyonlinelibrary.com]

The present study was performed at the Oulu University Hospital, Finland (Fig. 1A), and primarily consisted of a chart review of all relevant cases in the hospital's catchment area over a study period of five decades (1970 to 2022). The tertiary catchment area consists of all four central hospitals of Northern Finland and geographically covers 51% of Finland. In 2022, the total population of this territory was 730,664, and the population under the age of 16 years was 140,672.^10^ The Child Neurology Unit of the Oulu University Hospital, Finland, has approximately 6000 outpatient visits and 600 inpatient admissions per year.

The aims of this project were to define the epidemiological, clinical, neuroradiological, and genetic characteristics of PCDs to develop diagnostics and provide natural history data of PCDs that are critical for the development of future treatment approaches. We hypothesized that genetic etiologies belonging to the FDH were more common in the study region compared to the PCDs that are prevalent in other European populations.

### PEDIATAX Cohort

We established the PEDIATAX cohort by retrieving all case records at the Oulu University Hospital from 1970 to 2022 (manual records before the year 2000; electrical records from the year 2000 onwards; diagnosis <16 years) containing any of the *International Classification of Diseases* (ICD)‐9 or ICD‐10 codes that pertain to PCDs (the retrospective study arm) (Table S1). Newly referred patients with suspected or confirmed PCDs were recruited by attending physicians during the active phase of the project (between 2019 and 2022) to ensure systematic coverage of all patients with PCDs in our study region (the prospective study arm). We recorded information on diagnostic tests (including testing for Friedreich's ataxia in cases where the initial exome sequencing was inconclusive), preliminary neuroradiological data, all diagnoses, and disease courses. The clinical significance of identified genetic variants was assessed by SIFT, PolyPhen, and CADD,^11^, ^12^ and compared against information in the ClinVar, gnomAD (GRCh37, version 2.1.1), and Ensembl databases. In suspected encephalitis, routine investigations included brain MRI followed by etiological analyses of the cerebrospinal fluid, serum, and respiratory track samples (ie, polymerase chain reaction [PCR] of common CNS and respiratory pathogens). In cerebral palsy, genetic testing was conducted when the clinical presentation, history, or other findings raised suspicion of genetic etiology. PCDs were classified into the following categories based on etiology: non‐genetic etiology, definitive genetic diagnosis, variant of unknown significance (VUS), and undefined etiology. All personal identifying data were removed during data collection.

Brain imaging data (ie, images and radiological reports) were collected from the picture archiving and communication system (PACS) of the Oulu University Hospital, and by contacting all central hospitals in Northern Finland within the tertiary catchment area of the Oulu University Hospital. Data were analyzed systematically by a junior radiologist (S.H.) using a structured data sheet (Table S2). Initially, all images were analyzed blinded (ie, without prior knowledge of the underlying condition), and then re‐analyzed for typical neuroimaging findings of specific genes/conditions. Though the focus was on posterior fossa findings, we collected data on other abnormalities and incidental findings. Finally, all available images were re‐read by a consultant pediatric neuroradiologist (M.S.‐P.), and a consensus was reached.

The PEDIATAX cohort flowcharts are presented in Figures 1B and S1. Data collection sheets are provided in Tables S1 and S2.

### Literature Cohort

To enable the assessment of genotype‐to‐phenotype concordance between the PEDIATAX cohort and previously published cases, we created a comparative literature cohort by searching the PubMed database for published cases of each of the monogenic PCDs found in the PEDIATAX cohort. Our literature cohort was limited to genes whose variants in the PEDIATAX cohort had been classified as pathogenic according to the ACMG criteria.^13^ The literature search was performed in 2023. The search terms included the main and alternative titles of the disease (referencing the OMIM database) and the causal gene. Articles were included based on the following criteria: Age of onset <15 years of age; the phenotype was neurological and sufficiently described; the causal genetic variant was identified; and the article was in English and accessible via the library of the University of Oulu. Although an exact age of onset was not always available, we included all cases in which the first symptoms occurred <15 years of age. Cases where a genetic test was not performed for the participant, even if they were related to a second, genetically proven case, were excluded. We collected variant details, clinical features manifesting <15 years of age, and brain imaging results when available.

### Statistical Methodology

The cumulative incidence of PCDs was calculated using the Dx Method^14^ by dividing the number of observed cases by the number of live births (obtained from Statistics Finland^10^) during the study period (1970–2022). The trend in cumulative incidence was assessed using linear regression analysis.

### Ethical Approval

The PEDIATAX project is a single‐center registry study (205/2019, the Northern Ostrobothnia Hospital District). The results were anonymized according to the Finnish Act of the Secondary Use of Health and Social Data, that is, presenting those results with a frequency of fewer than three individuals per variable with “<3,” instead of the exact number, and removing all IDs of individuals retrieved via the registry study. The anonymity of the results was verified by the Finnish Social and Health Data Permit Authority “Findata,” including the removal of facial features from brain MRI. Informed consent by signed consent documents was obtained for individuals whose MRIs were included based on the PEDIATAX research protocol approved by the regional ethics committee (EETTMK: 67/2019, amendments October 30, 2020, and April 19, 2021) and conducted in accordance with the Declaration of Helsinki.

## Results

### Epidemiology of PCDs in Northern Finland

In total, we identified 107 cases with a PCD (ie, the PEDIATAX cohort) (Fig. 1C). Over the study period, the cumulative incidence of PCDs was 21.9 per 100,000 live births, with a 10.1 (*P* = 0.027) increase within each year category (Table 1). Demographic and epidemiological data in the PEDIATAX cohort are shown in Table 2.

**TABLE 1:** The cumulative childhood incidence of pediatric cerebellar disorders and the number of live births in the study region (unless otherwise stated) by decade, calculated using the Dx Method^14^

	1970–1979	1980–1989	1990–1999	2000–2009	2010–2019	2020–2022	β value (p)^a^	1970–2022
PCD cases observed	7	25	18	18	26	13	**10.1 (0.027)**	**107**
Live births^b^	92,393^c^	107,886	97,348	89,161	82,127	19,753		**488,668**
Cumulative childhood incidence per 100,000 live births	7.6	23.2	18.5	20.2	31.7	65.8		**21.9**

Abbreviation: PCD, pediatric cerebellar disorder.

^a^ Linear regression analysis.

^b^ Obtained from Statistics Finland.

^c^ Oulu and Lapland provinces.

**TABLE 2:** Demographic information, developmental history, age at disease onset and diagnoses in the PEDIATAX cohort

	All PCDs (n = 107)	Genetic (n = 46)	Acquired (n = 13)
Demographic information			
Age at end of study period^a^, y, median (range)	28 (2 to 61)	20 (2 to 59)	41 (3 to 55)
Deceased	7 (6.5)	5 (11)	0 (0,0)
Age at death, y, median (range)	18 (5 to 54)	18 (5 to 54)	NA
Male sex (%)	56 (52)	20 (44)	6 (46)
Developmental history			
Age when symptoms were observed^b^, mo, median (range)	12 (0 to 144)	14 (0 to 120)	24 (0 to 144)
Motor developmental delay	86 (80)	35 (76)	6 (46)
Age at PCD diagnosis, mo, median (range)	41 (0 to 430)	48 (0 to 430)	36 (6 to 161)
Diagnoses			
Movement disorder	95 (89)	42 (91)	13 (100)
Ataxia	76 (71)	34 (74)	12 (92)
Tremor	30 (28)	10 (22)	3 (23)
Dystonia	15 (14)	11 (24)	<3^3^
Athetosis	18 (17)	8 (17)	<3^3^
Intellectual disability	32 (30)	18 (39)	0 (0,0)
Mixed specific developmental disorders (F83)	10 (9.3)	4 (8,7)	0 (0,0)
Seizures	33 (31)	19 (41)	<3^c^

*Note*: Data are presented as n (%)/cases with available information, unless otherwise stated.

Abbreviations: y, years; mo, months; NA, not applicable; PCD, pediatric cerebellar disorder.

^a^ December 31, 2022.

^b^ Observed either by a primary caretaker or an attending clinician. Five cases lacked exact data on age. In these cases, the observed age was estimated to the nearest year.

^c^ The results were anonymized according to the Finnish Act of the Secondary Use of Health and Social Data, that is, presenting those results with a frequency under three individuals per variable with “<3,” instead of exact number, and removing all IDs of individuals retrieved *via* the registry study.

### Etiologies of PCDs in Northern Finland

The etiologies of PCDs are presented in Figure 1C. The etiology was defined in 59 (55%) cases: 39 (66%) were monogenic, seven (12%) chromosomal, and 13 (22%) non‐genetic. The diagnostic yield of NGS (ie, targeted panels, exome sequencing, or genome sequencing) among individuals with ataxia (n = 76) was 65%. The etiologies of the 39 monogenic cases included genes in the following categories (Fig. 1D): trinucleotide repeat disorders, lysosomal disorders, mitochondrial diseases, other metabolic disorders, pediatric neurotransmitter diseases, ion channel related disorders, and other genetic disorders. A variant of unknown significance (VUS) was found in four (4%) cases, including genes *KIF1C, RORA*, and *SPTBN2*.

The non‐genetic etiologies (n = 13) included postinfectious ataxia, cerebellitis, cerebral palsy, encephalitis, and hypoxic–ischemic brain injuries. Immunological tests (including white blood cell counts and immunoglobulin levels) were normal in all tested patients with non‐genetic etiologies. The remaining 45 cases consisted of individuals with undefined etiologies (42%) despite initial investigations, of cases where a VUS was found, and of older cases in which no etiological studies had been performed.

Certain hereditary disorders are more prevalent in Finland than in other parts of the world because of small founder populations, bottleneck events, and other historical factors. This phenomenon is known as the Finnish Disease Heritage (FDH).^15^ Three distinct FDH diseases were present in nine cases of the PEDIATAX cohort: epilepsy, progressive myoclonic, type 1 (EPM1, *CSTB* with the Finn Major variant CCC‐CGC‐CCC‐GCG repeat expansion, minor allele frequency [MAF] not available); Salla disease (*SLC17A5*, including Finn Major variant c.115C>T, MAF in Finns 0.005911, MAF in non‐Finnish Europeans 0.0006116); and infantile‐onset spinocerebellar ataxia (IOSCA, *TWNK*, including cases of homozygous variants and cases of compound heterozygous variants with Finn Major variant c.1523A>G, MAF in Finns 0.002866, MAF in non‐Finnish Europeans 0.00003096).^9^ These cases accounted for 8.4% of all etiologies in this study, and 23% of monogenic etiologies. Detailed variant data are shown in Table S3.

### Age of Onset in PCDs and Genotype Concordance in the Literature Cohort

We defined age of onset as the age at which each patient's first symptoms or signs related to a PCD were either reported by guardians or recorded upon clinical examination by a physician. In the PEDIATAX cohort, the median age of onset was 1 year (range: 0–12 years) for all cases of PCD (Table 2). Median age at diagnosis was 3.4 years, with a range from birth to the age of 36 years.

**FIG. 2:**
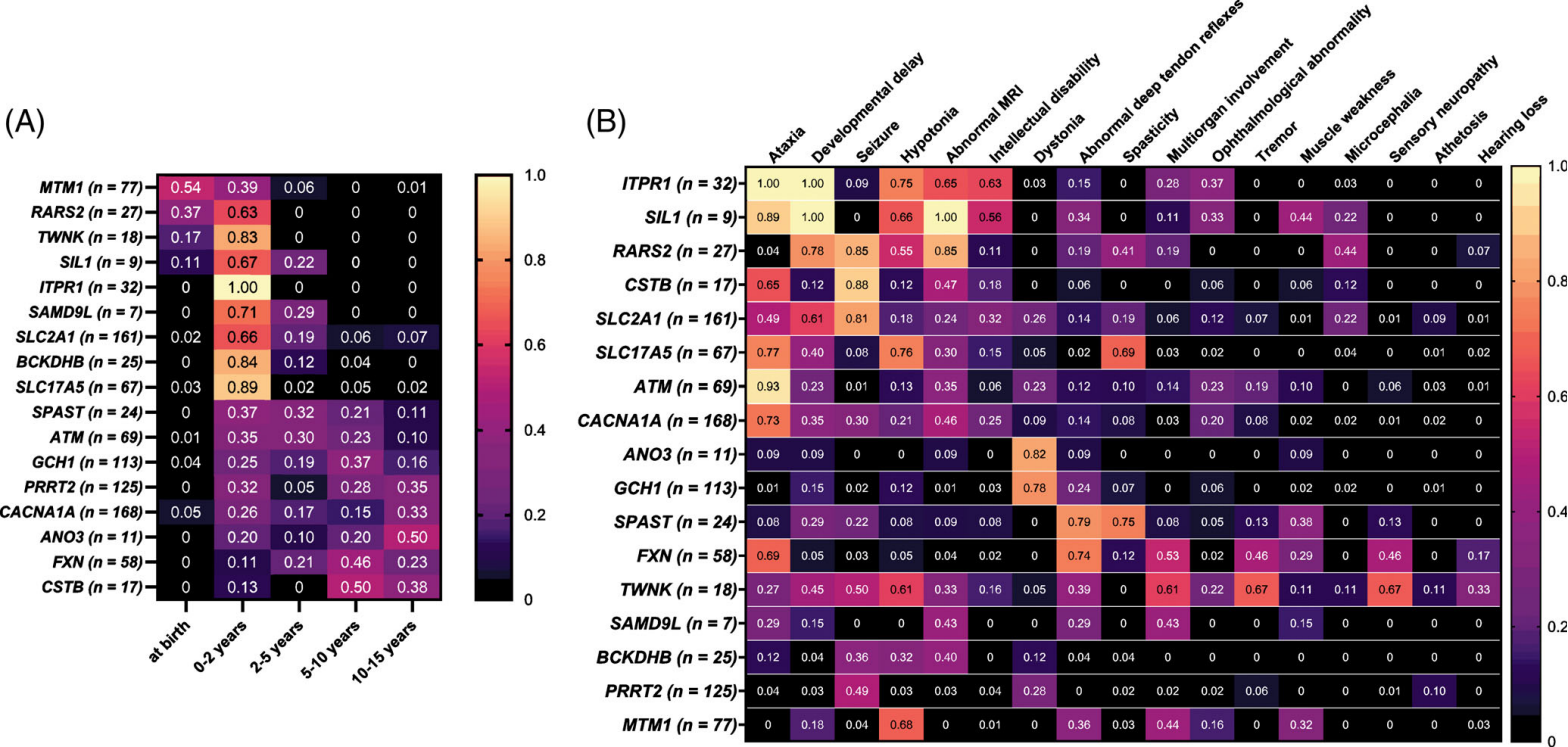
Age at onset in genetically confirmed PCDs (**A**) and a heatmap of genotype‐to‐phenotype correlations (**B**) in previously published cases (n = 1007). The proportion of patients with the indicated age of onset or phenotype per genotype is present as dark violet (0) to light yellow (1.0). PCD, pediatric cerebellar disorder. [Color figure can be viewed at wileyonlinelibrary.com]

The literature review identified 1007 clinical cases from 535 published case reports that featured one of the 17 distinct genes with pathogenic variants in the PEDIATAX cohort.^13^ These are presented in order of age of onset in Figure 2A, which shows that genetic PCDs with very early onset (from birth until age 2 years) included those caused by variants in the *MTM1, RARS2, TWNK*, *SIL1*, and *ITPR1* genes; those with onset during the first 5 years of age included the *SAMD9L*, *BCKDHB, SLC17A5*, and *SLC2A1* genes; and monogenic PCDs with the widest spectrum of age of onset included the *SPAST*, *ATM*, *PRRT2*, *CACNA1A*, *GCH1*, *ANO3*, *FXN*, and *CSTB* genes, with onset extending from the very early (0–2 years) to the late (10–15 years) categories (Fig. 2A). In the PEDIATAX cohort, the pattern of onset was broadly the same as in the published cases except for three gene categories where age of onset was reported later than previously observed (*MTM1*, *RARS2*, and *SAMD9L*). References for the literature cohort are listed in File S3.

### Clinical Features of PCDs

Diagnoses in the PEDIATAX cohort are reported in Table 2. Motor developmental delay was observed in 86 (80%) cases. Movement disorders manifested in 95 (89%) of all cases. Ataxia was present in 76 (71%), classified as non‐progressive in 46 (61%), progressive in 22 (29%), and varying in eight (11%) cases. Among the genetic etiologies, 34 (74%) of cases had ataxia, out of which 17 (50%) were non‐progressive and 17 (50%) were progressive or varying. The genes associated with progressive ataxia were *ANO3, ATM, CACNA1A, CSTB, FXN, MTM1, RARS2, SAMD9L, SLC17A5*, and *TWNK*. Among non‐genetic etiologies, ataxia manifested in 12 (92%) cases and was mostly non‐progressive (n = 10). Tremor was observed in 30 (28%) cases (n = 10 for genetic etiologies; n = 3 for non‐genetic etiologies). Progressive tremor was associated with genes *CACNA1A*, *CSTB*, and *SAMD9L*. Dystonia was present in 15 (14%) cases (n = 11 for genetic etiologies; n < 3 for non‐genetic etiologies) and athetosis in 18 (17%) cases (n = 8 for genetic etiologies; n < 3 for non‐genetic etiologies). Progressive dystonia was associated with *ANO3* and a microdeletion in Xp11.4 including the *CASK* gene, whereas progressive athetosis was seen with *ATM* and *TWNK*.

Epilepsy was diagnosed in 33 (31%) of all PCDs, out of which 19 cases had a genetic etiology for their PCD. Six individuals with epilepsy presented with probably epileptogenic cerebral abnormalities in brain MRI, whereas six presented with malformations restricted to the cerebellum, accompanied by non‐epileptogenic cerebral abnormalities (ie, ventricular enlargement, slight leukomalacia, slightly small hippocampi or corpus callosum, microcephaly) in some individuals.

The most frequent clinical features among the study and literature cohorts were ataxia, developmental delay, seizures, hypotonia, and abnormality in brain MRI, although these phenotypes were remarkably variable (Fig. 2B). Clinical findings that were associated with fewer genetic etiologies were hearing loss, sensory neuropathy, and microcephalia (Fig. 2B). The genotype‐to‐phenotype associations in the PEDIATAX cohort were mainly in line with those seen in the literature cohort, except for 10 gene categories. The features that were more common in the PEDIATAX cohort than in the literature cohort included athetosis, hypotonia, spasticity, and ophthalmological findings with *ANO3*; developmental delay and epilepsy with *FXN*; ataxia and spasticity with *GCH1*; ataxia with *MTM1*; muscle weakness with *RARS2*; microcephaly and tremor with *SLC2A1*; multiorgan involvement, intellectual disability, and seizures with *SLC17A5*; ataxia and multiorgan involvement with *SPAST*; and intellectual disability, ophthalmological findings, and hearing loss with *TWNK*. Furthermore, the features that were common among previously reported cases but were absent in the PEDIATAX cohort included dystonia with *ATM*; and spasticity, dystonia, abnormal reflexes, and intellectual disability with *SLC2A1*. The case histories of six PEDIATAX participants (*ITPR1*, *SAMD9L*, and Xq11–q12 microdeletion) have been previously published.^16^, ^17^, ^18^, ^19^

### Neuroradiology in Early‐Onset PCDs

**FIG. 3:**
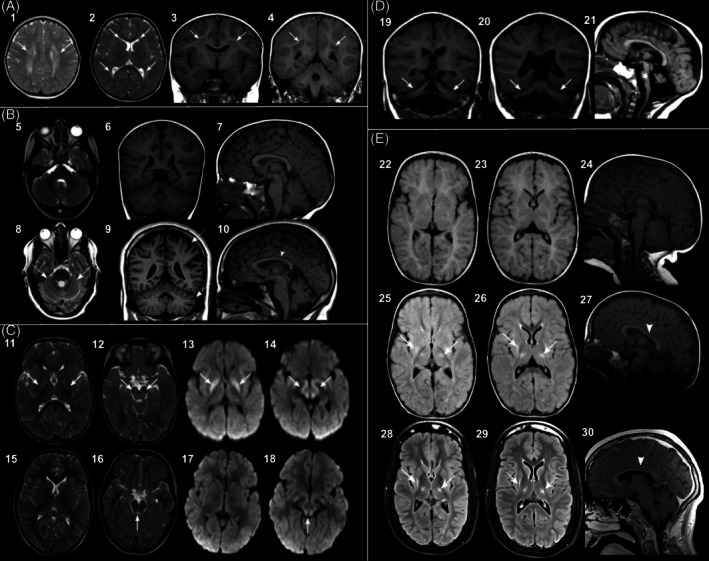
Examples of neuroradiological findings in the PEDIATAX cohort. (**A**) Microduplication 15q13.3 (1 year 7 months): Symmetrical periventricular and deep white matter signal changes (arrows) in axial T2 (high signal in 1, 2) and coronal T1 flair (low signal in 3, 4) images. (**B**) *SPAST* gene: At 3 years and 9 months (5–7) MRI was considered normal. At 18 years, MRI shows progressive cerebral and cerebellar atrophy (arrows, 8, 9), slight ventricular enlargement, and thinner corpus callosum (arrowhead 10). (**C**) Maple syrup urine disease, *BCKDHB* gene: During infection at 1 year and 10 months globus pallidus, cerebral peduncles and dorsal brainstem showed bilateral abnormal high T2 signal (arrows, 11, 12) and diffusion restriction (arrows 13, 14) suspicious of metabolic etiology. During infection at 10 years and 3 months MRI showed only slight T2 signal increase (arrow, 16) and slight diffusion restriction (arrow, 18) around the cerebral aqueduct and the earlier findings had normalized (15–18). (**D**) Microdeletion Xp11.4 (9 months): Severe pontocerebellar hypoplasia with a typical dragon fly sign (arrows) in coronal images (19, 20). Both cerebellar hemispheres and vermis are hypoplastic. Marked pontine hypoplasia (star) is demonstrated in the sagittal plane (21). (**E**) Cerebral palsy: At 5 months brain MRI was considered normal in a child with diagnosis of cerebral palsy (22–24). At 2 years and 6 months there were signs of Wallerian degeneration in the corticospinal tracts and atrophy and increased T2 signal bilaterally in thalami and nuclei lentiformis (arrows 25, 26) and around the posterior horns of lateral ventricles. In addition, corpus callosum was thinner than normally (arrowhead 27). The findings persisted at 17 years (28–30).

Neuroradiological findings in the PEDIATAX cohort are presented in Table 3. Altogether 49 patients were included for neuroradiological analysis consisting of 38 cases with brain MRI scans, four cases with computed tomography (CT) scans, and seven cases with previous high‐quality reports. Of these, 32 (65%) had abnormal brain imaging findings; five examples are shown in Figure 3. Cerebellar atrophy, the most common finding in the PEDIATAX cohort, was classified as progressive in seven cases based on serial scans. These patients had causal variants in *FXN*, *CACNA1A*, *CYB5R3*, *SPAST* (Fig. 3B), and *SAMD9L*. Of note, three patients were found to have pathogenic variants in the *ATM* and *SAMD9L* genes but did not display evidence of cerebellar atrophy at the time of available brain MRI scans. Progressive cerebellar atrophy was associated with progressive ataxia only in four cases (40%; *ATM*, *CACNA1A*, *FXN*, and *SAMD9L*). Non‐progressive cerebellar atrophy was found with causal variants in *SIL1* and *MTM1*. Posterior fossa abnormalities were found in 20 cases, and additional neuroradiological findings in 12 cases. Novel or rarely reported neuroradiological findings were associated with the following genes: Pontocerebellar hypoplasia (*ACTB*), progressive cerebellar atrophy without hypoplasia of pons and vermis (*CYB5R3*), cerebellar atrophy (*MTM1*), progressive cerebellar atrophy accompanied with delayed myelination within the centrum semiovale and corona radiata (*SPAST*).

**TABLE 3:** Neuroradiological findings in the PEDIATAX cohort

Etiology	Posterior fossa finding	Additional findings
*ACTB*	Pontocerebellar hypoplasia and a large posterior fossa cyst	Enlarged ventricles and a thin corpus callosum
*BCKDHB*	‐	Bilateral high T2 signal, and diffusion restriction in the globus pallidus, cerebral peduncles, and dorsal brainstem upon the first infection‐related Maple syrup urine disease (MSUD) episode, including central nervous system symptom manifestation—the second MSUD episode was associated with slight T2 signal increase and diffusion restriction around the cerebral aqueduct, while the earlier findings had normalized
*ATM*	Based on genetic diagnosis, no serial scans: progressive cerebellar atrophy	‐
*CACNA1A*	Progressive cerebellar atrophy	‐
*CASK*	Pontocerebellar hypoplasia indicated by the dragon fly sign	Microcephaly (head circumference SD >3) and underdeveloped cerebrum
*CYB5R3*	Progressive cerebellar atrophy, small posterior fossa, hemorrhage among tentorium	Enlarged Cerebrospinal fluid (CSF) spaces, thin corpus callosum, an unusually small amount of white matter
*DDX3X*	Slight hypoplasia within lower side of the vermis, enlarged 4th ventricle	A thin corpus callosum, enlarged posterior horns of the lateral ventricles as well as 3rd ventricle
*ITPR1*	‐	Unspecific high‐signal intensities next to the anterior horn of the right lateral ventricle and in the frontal white matter, as well as a small arachnoid cyst, and possible residuals of a plexus hemorrhage
*FLNA*	Mega cisterna magna	‐
*FXN*	Progressive cerebellar atrophy	‐
*KIF1A*	Arachnoid cyst in posterior fossa	‐
*MTM1*	Non‐progressive cerebellar atrophy	‐
*SAMD9L*	Progressive cerebellar atrophy	‐
*SIL1*	Non‐progressive cerebellar atrophy	‐
*SLC17A5*	Vermian atrophy	Slightly delayed myelination, hypomyelination (diffuse or severe and progressive), a very thin corpus callosum, brachycephaly
*SPAST*	Progressive cerebellar atrophy	Delayed myelination within the centrum semiovale and corona radiata
Microduplication, 15q13.3.	‐	Symmetric periventricular and deep white matter signal changes in axial T2 and coronal T1 flair images
Microdeletion, 13q32.2q32.3.	‐	Alobar holoprosencephaly
Microdeletion Xq11–q12.	‐	Enlarged CSF space in posterior fossa
Microdeletion at Xp11.4 (606 kB including the *CASK* gene)	Severe pontocerebellar hypoplasia	‐
Cerebral palsy	Non‐progressive vermian atrophy, a small posterior fossa	‐

The imaging findings among non‐genetic etiologies included Wallerian degeneration in the corticospinal tracts and atrophy and increased T2 signal bilaterally in the thalami and posteromedial nuclei lentiformis, in addition to a thin corpus callosum shown in Figure 3B (dyskinetic cerebral palsy due to birth asphyxia); a small posterior fossa with slight vermian atrophy (cerebral palsy); slight periventricular leukomalacia possibly due to preterm birth (cerebellitis); unspecific asymmetry within the frontal area of the ventricles, and slight ventricular enlargement (cerebellitis); and leptomeningeal enhancement in the posterior fossa, a single small microhemorrhage in the cerebellum, and slight diffusion restriction in the splenium of the corpus callosum (adenoviral meningoencephalitis).

Brain MRI scans were unremarkable for the following 17 participants: three with postinfectious ataxia, one with ataxia after drowning, one with microduplication in 17q12, and 12 with causal variants in genes *ANO3*, *CSTB*, *GCH1, GNAO1*, *KCNA1*, *PRRT2*, *RARS2*, *SLC2A1*, and *TWNK*. The collection of imaging data is shown in Figure S1, and the detailed characteristics of the imaging data are presented in File S1.

## Discussion

We present data on PCDs in Northern Finland over a 50‐year period (1970–2022). In total, our cohort included 107 cases, giving a cumulative incidence of 21.9 per 100,000 live births. Our data replicate previous findings that PCDs as a disease group are a “common” rare disorder, although individual PCDs can be ultra‐rare.^20^ Interestingly, the cumulative incidence in the present study was higher toward the later decades. We suggest that this increase has resulted from improved clinical awareness of PCDs and advances in diagnostic methods, mostly the availability of NGS and improvements in neuroimaging technologies especially during the last decade.^7^ Importantly, our data demonstrate a high proportion of genetic etiologies in early‐onset ataxias.

Our findings show that PCDs are considerably heterogeneous in phenotype (Fig. 2). Based on our review of genotype‐to‐phenotype correlations in the PEDIATAX cohort and a literature cohort of 1007 published cases, the most frequent clinical manifestations were ataxia, developmental delay, seizures, hypotonia, and abnormality in brain MRI, which trigger suspicion of a genetic disorder but do not distinguish between different genetic etiologies (Fig. 2). Clinical manifestations associated with fewer genotypes were hearing loss, sensory neuropathy, and microcephalia, which could assist in the interpretation of NGS data by narrowing down the list of candidate genes. However, the phenotypic variability within defined PCDs was remarkable, as demonstrated previously with adult‐onset non‐expansion spinocerebellar ataxias.^21^ In addition, genotype‐to‐phenotype interpretation is still limited by the low number of published cases in some ultra‐rare genetic etiologies. Overall, phenotypes ranged from transient symptoms (associated with the *BCKDHB* variant) to persistent but mild symptoms (for example, those driven by *SAMD9L* variants) to severely affected individuals with various etiologies. The most consistent diagnostic principle appeared to be the age of onset, with distinctions between the very early (birth to 2 years), early (up to 5 years), and late (5 to 15 years) age categories. The median age at onset was 1 year with a considerable range (0–12 years), whereas the median age at diagnosis was 3.4 years, reflecting a diagnostic delay of several years that is further confounded by heterogenous phenotypes. The genotype‐to‐phenotype correlations we found in the PEDIATAX cohort were consistent with those in the published literature, except for specific novel findings in 10 gene categories. These data reflect the unique genetic landscape of the study area and could aid in developing the diagnostics of PCDs in Northern Finland by revealing precise characteristics of the study population.

Although ataxia was, as expected, the most common movement disorder in our PCD cohort (71%), tremor was present in 28% of individuals and athetosis and dystonia in 17% and 14%, respectively, indicating that non‐ataxia disorders are not uncommon in the PCD population. This finding aligns with that of a recent retrospective cohort study of 51 individuals with pediatric movement disorders, which found ataxia in 53% of cases, dystonia in 49%, tremor in 16%, and chorea‐athetosis in 13%.^22^ Another recent study including 606 patients with a childhood‐onset movement disorder found tics to be the most common diagnosis (57%), whereas dystonia was the most common non‐tic movement disorder (12%) in contrast with our findings.^23^ Additionally, 3% had ataxia, 3% chorea, 2% spasticity, and 2% tremor.^23^ Notably, many participants in the PEDIATAX cohort had combinations of two or more movement disorders, which was another common observation in previous studies.^1^, ^22^ Nevertheless, it is becoming evident that the cerebellum plays a significant role in the pathogenesis of non‐ataxia movement disorders such as dystonia and paroxysmal kinesigenic dyskinesia^24^, ^25^ potentially redefining what constitutes a cerebellar disorder. In the future, standardized minimal requirements are needed to ensure systematic reporting of clinical phenotypes in PCDs. Similarly, the use of standardized scoring systems such as the Scale for the Assessment and Rating of Ataxia (SARA; applicable from 8 years of age),^26^ which we have not included in the current study, will be necessary to facilitate clinical trials.

The specific focus of the PEDIATAX study was on genetic etiologies, specifically monogenic PCDs: we identified 24 monogenic etiologies from 39 individuals among our 107 patients. The diagnostic yield for NGS studies among patients with ataxia was 65%; the overall diagnostic yield was 55%. Importantly, the list of genes associated with PCDs is expanding, with *DDX3X* and *GNAO1* as recent examples.^27^, ^28^, ^29^, ^30^ Moreover, cerebellar manifestations are being linked to previously described disorders, for example, developmental and epileptic encephalopathy (*CACNA1A*), and methemoglobinemia type 2 (*CYB5R3*).^31^, ^32^ Currently, one of the major questions in the field is how monogenic etiologies can underlie such different phenotypic presentations.

Seven of the monogenic disorders identified in our cohort had targeted treatment options available (*SLC2A1*, OMIM*138140; *BCKDHB*, OMIM*248611, *GCH1* OMIM*600225, *ANO3* OMIM*610110, *CACNA1A* OMIM*601011, *PRRT2* OMIM*614386, and *SAMD9L* OMIM*611170). However, the development of new treatment options beyond symptom‐alleviating management is becoming more complicated as the list of identifiable etiologies and potential treatment targets for monogenic PCDs grows.^1^, ^33^ To overcome this challenge, we suggest that efforts should be refocused to tackle shared disease mechanisms according to common PCD etiologies.^1^, ^34^, ^35^ Although the ultimate cure of monogenic disorders would eventually require fixing their underlying genetic defects (ie, precision medicine), in many cases disease‐modifying therapies do exist and, when utilized with accurate timing, can improve the neurological outcome and prevent progression.^33^, ^36^, ^37^ Thorough characterization of genotype‐to‐phenotype correlations is pivotal for uncovering potential biomarkers to enable early interventions and monitoring treatment outcomes.^33^, ^38^

Neuroimaging is a central biomarker in PCDs and is essential for diagnostics and monitoring. We provide some examples of brain MRI findings associated with defined etiologies in the PEDIATAX cohort (Fig. 3). The most common neuroimaging finding in the PEDIATAX cohort was cerebellar atrophy that behaved in line with the literature. Although cerebellar atrophy is a frequent finding in genetic cerebellar ataxias,^3^ it is nonspecific and has only limited diagnostic value as an isolated finding.^39^ The specificity of diagnostic imaging of cerebellar hypoplasia and atrophy has developed during recent years; we have advanced from defining many posterior fossa findings as Dandy‐Walker malformations or as part of the Dandy‐Walker continuum to more detailed reporting. Recently, although Whitehead et al (2022) redefined the imaging definition of Dandy‐Walker malformation, they observed misdiagnoses in almost a quarter of previous reports.^40^ Thus, it will be essential to pursue a more detailed description of posterior fossa imaging findings to enable the recognition of previously unnoticed phenotypes that are linked to certain genetic conditions. Close collaboration between imaging and genetic research is necessary to enable more specific clinical diagnostics.

One limitation of the current study pertains to reporting bias in the literature review. We consider the clinical features included in our review to be common to PCDs, and information on the presence or absence of these features was present in most articles. However, some clinical features were not discussed in each article, possibly contributing to reporting bias (ie, underestimation of the frequency). All features were systematically assessed within the PEDIATAX cohort. Another limitation of the study is the small number of cases of non‐genetic etiologies, some of which may have been missed in the register search due to a lack of standardized ICD‐10 codes. ORPHAcodes were introduced in early 2025 as part of our electronic medical record system to improve the searchability of the records. In addition, a separate study of etiologies of cerebral palsy is currently ongoing in the study region and may yield additional insight into non‐genetic ataxias.

In conclusion, our data highlight the considerable phenotypic heterogeneity associated with PCDs but further suggest age of onset, hearing loss, sensory neuropathy, and microcephalia as practical clinical clues in distinguishing between individual PCDs. Our dataset provides a thorough perspective on PCDs, expanding the clinical spectrum of these rare disorders and aiding clinicians in recognizing this remarkable group of movement disorders, their most common comorbidities, and etiologies. Finally, we provide a basis for future research on diagnostic approaches, disease mechanisms, and novel treatment targets, calling for further data on the natural history of this complex disease entity for future clinical trials.

## Author Roles

(1) Research Project: A. Conception, B. Organization, C. Execution, D. Supervision; (2) Data acquisition: A. Patient recruitment, B. Execution; (3) Statistical Analysis: A. Design, B. Execution, C. Review and Critique; (4) Manuscript Preparation: A. Writing of the First Draft, B. Review and Critique.

K.G.: 1A, 1C, 2B, 3A, 3B, 4A, 4B.

S.H.: 1C, 2B, 4A, 4B.

J.E.: 1C, 2B, 4A, 4B.

T.P.: 3A, 3B, 3C, 4B.

S.M.K.: 2B, 4B.

J.M.: 4B.

H.H.: 2A, 4B.

H.K.: 2A, 4B.

J.K.E.: 2A, 4B.

P.V.: 2A, 4B.

E.R.: 2A, 4B.

R.G.: 2A, 4B.

M.H.: 2A, 4B.

T.S.R.: 2A, 4B.

R.H.: 1B, 4B.

M.S.‐P.: 1D, 2B, 4B.

J.‐P.T.: 1A, 1B, 1C, 1D, 3C, 4A, 4B.

J.U.: 1A, 1B, 1C, 1D, 3C, 4A, 4B.

## Supporting information


**File S1.** Neuroradiological findings in PEDIATAX cohort.
**Tables S1–S2.** Data collection sheets.
**Figure S1.** Imaging data collection.

**File S2.** Supplementary File S2.

**File S3.** A list of articles included in the review of the literature.

## Data Availability

All data that can be shared openly are available with this article and supplementary files. Unpublic data include precise details about human participants and cannot be shared openly in their entirety. The corresponding author (J. U.) may make the complete dataset available upon receipt of a well‐founded request.
