# Supplementary material for: Phenotypic Heterogeneity in Genetic and Acquired Pediatric Cerebellar Disorders
Source: Mov Disord. 2025 May 6;40(9):1851–62. doi: 10.1002/mds.30210 (PMC12485580; doi:10.1002/mds.30210)
Supplement: Supplementary file 1 — File S1. Neuroradiological findings in PEDIATAX cohort. Tables S1–S2. Data collection sheets. Figure S1. Imaging data collection. [file MDS-40-1851-s001.docx]

**Supplementary File 1**

**Phenotypic heterogeneity in acquired and genetic pediatric cerebellar disorders**

**Granath, K. *et al*.**

**Supplementary information S1; Supplementary Tables S1-S2; Supplementary Figure S1**

**SUPPLEMENTARY INFORMATION S1: NEURORADIOLOGICAL FINDINGS IN THE PEDIATAX COHORT**

Of all cases with available imaging data (n=49), 38 had at least one brain MRI and four had CT images only; 13 had both MRI and CT images; and 7 had reliable reports only. Of the MRI data, three cases had only film images and the other 35 digital images. Twenty-four cases had more than one brain MRI scan and seven had more than one CT scan. Including all subjects with reliable imaging data or reports, subjects were imaged on average 1.9 times with MRI and 0.7 times with CT. For 14 subjects, follow-up images were captured at least 2 years apart.

Including all subjects (N=49), the mean age at first brain imaging was 7 years and 4 months. The mean age for first brain MRI was 7 years and for CT, 16 years and 1 month. However, most subjects were imaged during infancy and childhood. For 22 subjects, the first images were acquired during infancy (before age 2 years at mean age 11 months), 15 during childhood (between 2 to 12 years at mean age 4 years), and <3 during adolescence (between 12 to 18 years at mean age of 13 years and 10 months. For nine cases there were only images from adulthood (after 18 years first images at mean age of 27 years).

Of all subjects, 17 had no significant brain imaging findings and the images were reported as normal. Of those with only reliable reports available, <3 CT scans were reported as normal without evident pathology, and <3 had only slight ventricular and cortical cerebrospinal fluid (CSF) space enlargement without an apparent reason. Of the MRI reports, four were reported as normal, and <3 subjects had repeated normal findings. From the available imaging data, of the subjects with no initial findings based on primary reports, only <3 were found to have mild cerebellar atrophy in the thorough re-review process.

**SUPPLEMENTARY TABLES AND FIGURES**

**Supplementary Table S1.** Data collection sheet, clinical data, and SPSS values.

| birth date | dd.mm. yyyy |
| --- | --- |
| deceased, date | dd.mm. yyyy |
| ICD-9 and ICD-10 codes, used at the initial collection of patients before screening for included patients. | 2775B, 3331A, 334 (3340, 3341, 3342, 3343, 3344, 3348, 3349), 34799, 3483X, 7812A  G11, G11.0, G11.00, G11.01, G11.02, G11.08, G11.10, G11.11, G11.12, G11.13, G11.14, G11.18, G11.2, G11.3, G11.4, G11.8, G11.9, Q03, Q03.1, Q07, Q87.83. |
| other included patients with suspected or confirmed paediatric cerebellar disorders, identified by attending physicians | 1= pathogenic variant (*BCKDHB)*  2= pathogenic variant (*CACNA1A)*  3= pathogenic variant (*CYB5R3)*  4= pathogenic variant (*SAMD9L*)  5= pathogenic variant (*SLC2A1*)  6= likely pathogenic variant (*DDX3X*)  7 = likely pathogenic variant (*GNAO1*)  8= likely pathogenic variant (*PRRT2*)  9= chromosomal aetiology (15q13.3 microduplication)  10= chromosomal aetiology (17q12 microduplication)  11= non-genetic aetiology (Adenoviral meningoencephalitis)  12 = variant of unknown significance (*KIF1C*)  13=variant of unknown significance (*RORA)*  14= Suspected PCD, aetiology unknown, |
| age when deceased | months (mo) |
| age when symptoms were first observed | mo |
| delayed motor development | mo |
| fine motorics | 0 = normal  1 = delayed  2 = regressive |
| gross motorics | 0 = normal  1 = delayed  2 = regressive |
| highest motor developmental milestone achieved, age | mo |
| hypotonia | mo  0 = normal  1 = non-progressive  2 = progressive  3=varying  4=undefined clinical course |
| spasticity | mo  0 = normal  1 = non-progressive  2 = progressive  3=varying  4=undefined clinical course |
| abnormal tendon reflexes | mo  0 = normal  1 = non-progressive  2 = progressive  3=varying  4=undefined clinical course  5=absent or diminished reflexes |
| tremor | mo  0 = normal  1 = non-progressive  2 = progressive  3=varying  4=undefined clinical course |
| ataxia | mo  0 = normal  1 = non-progressive  2 = progressive  3=varying  4=undefined clinical course |
| athetosis | mo  0 = normal  1 = non-progressive  2 = progressive  3=varying  4=undefined clinical course |
| dysdiadochokinesis | mo  0 = normal  1 = non-progressive  2 = progressive  3=varying  4=undefined clinical course |
| incoordination | mo  0 = normal  1 = non-progressive  2 = progressive  3=varying  4=undefined clinical course |
| peripheral neuropathy | mo  0 = normal  1 = non-progressive  2 = progressive  3=varying  4=undefined clinical course |
| social cognition | mo |
| intellectual disability | mo  ICD-10 code |
| language disorder, problems with speech | mo  explanation: producing speech, understanding speech, both |
| behavioral problems | mo |
| epilepsy | mo |
| vomiting | mo |
| head circumference at birth | cm, ±SD |
| latest head circumference | mo, cm, ±SD |
| nystagmus | mo  0 = normal  1 = non-progressive  2 = progressive  3=varying  4=undefined clinical course |
| ophtalmological symptoms | mo |
| hearing symptoms | mo |
| dermatological / cutaneous symptoms | mo |
| hepatomegaly | mo |
| splenomegaly | mo |
| heart symptoms | mo |
| kidney abnormality | mo |
| intestinal symptoms apart from vomiting | mo |
| endocrinological symptoms | mo |
| muskulosceletal symptoms | mo |
| anomalies | mo  specification |
| electrophysiolocical findings |  |
| ENMG, ENG | 0=normal  1=borderline  2=abnormal  4= not available |
| EEG | 0=normal  1=abnormal, focal  2=abnormal, generalized  3=abnormal, combined  4=other abnormal (specification)  5=not available |
| positive family history |  |
| sibling | 1=yes |
| parent | 1=yes |
| grandparent | 1=yes |
| other 2. or 3. level relative | 1=yes |
| laboratory tests | specification |
| etiological investigations | specification |
| Other features, specify | specification |

**Supplementary Table S2.** Neuroradiology data collection sheet.

| **SUBJECT NRO** | Finding | | Control imaging | Notes |
| --- | --- | --- | --- | --- |
| **Modality MRI (1) CT (2) US (3) and date** |  | |  |  |
| **Age at imaging (mo)** |  | |  |  |
| **Cerebellar myelination**  normal (0) |  | |  |  |
| delayed (1) |  | |  |  |
| missing (2) |  | |  |  |
| permanent hypomyelination (follow-up images at least 6 months after) (3) |  | |  |  |
| **Cerebellar hemispheres**  normal (0) |  | |  |  |
| agenesia (1) |  | |  |  |
| bilateral hypoplasia (2) |  | |  |  |
| unilateral hypoplasia (3) |  | |  |  |
| dysplasia (4) |  | |  |  |
| atrophy (5) |  | |  |  |
| agyria (6) |  | |  |  |
| pachygyria (7) |  | |  |  |
| lissencephaly (8) |  | |  |  |
| polymicrogyria (9) |  | |  |  |
| periventricular heterotopy (10) |  | |  |  |
| macrocerebellum (11) |  | |  |  |
| cerebellar cleft (12) |  | |  |  |
| **Vermis**  normal (0) |  | |  |  |
| agenesia (1) |  | |  |  |
| hypoplasia (2) |  | |  |  |
| Isolated inferior vermian hypoplasia (3) |  | |  |  |
| rhombencephalosynapsis (4) |  | |  |  |
| **Peduncles**  normal (0) |  | |  |  |
| abnormal signal (1) |  | |  |  |
| molar tooth sign (2) |  | |  |  |
| hypoplasia (3) |  | |  |  |
| **Cerebellar nuclei**  normal (0) |  | |  |  |
| abnormal signal (1) |  | |  |  |
| **Brainstem**  normal (0) |  | |  |  |
| hypoplasia (1) |  | |  |  |
| atrophy (2) |  | |  |  |
| pontine tegmental cap dysplasia (3) |  | |  |  |
| tectum dysplasia (4) |  | |  |  |
| **Medulla oblongata and spinal cord**  normal (0) |  | |  |  |
| syringomyelia (1) |  | |  |  |
| abnormal signal (2) |  | |  |  |
| **CSF spaces**  normal (0) |  | |  |  |
| ventricular dilatation due to hydrocephalus (1) |  | |  |  |
| ventricular dilatation due to cerebellar/brainstem atrophy (2) |  | |  |  |
| ventricular dilatation due to posterior fossa anomaly (3) |  | |  |  |
| dilated 4th ventricle (4) |  | |  |  |
| mega cisterna magna (5) |  | |  |  |
| Blake pouch cyst (6) |  | |  |  |
| posterior fossa arachnoid cyst(s) (7) |  | |  |  |
| dilated lateral ventricles without hydrocephalus (8) |  | |  |  |
| **Cerebral shunt**  no (0) |  | |  |  |
| yes (1) |  | |  |  |
| **Cerebellar cysts**  none (0) |  | |  |  |
| symmetric (1) |  | |  |  |
| asymmetric (2) |  | |  |  |
| **Cerebellar calcifcations**  none (0) |  | |  |  |
| symmetric (1) |  | |  |  |
| asymmetric (2) |  | |  |  |
| **White matter abnormality (cerebrum)**  none (0) |  | |  |  |
| hypomyelination (1) |  | |  |  |
| frontal (2) |  | |  |  |
| parietal (3) |  | |  |  |
| occipital (4) |  | |  |  |
| temporal (5) |  | |  |  |
| low volume (6) |  | |  |  |
| explanatory |  | |  |  |
| **Basal ganglia**  normal (0) |  | |  |  |
| T2 signal increase (1) |  | |  |  |
| atrophy (2) |  | |  |  |
| calcifications (3) |  | |  |  |
| **Mesencephalon**  normal (0) |  | |  |  |
| abnormal (1) |  | |  |  |
| **Corpus callosum**  normal (0) |  | |  |  |
| thin (1) |  | |  |  |
| hypoplastic (2) |  | |  |  |
| missing (3) |  | |  |  |
| T2 signal increase (4) |  | |  |  |
| **Gray matter abnormality (cerebrum)**  none (0) |  | |  |  |
| proliferation disturbance (microcephalia, megalencephalia) (1) |  | |  |  |
| migrational distrubance (e.g., heterotopy, lissencephaly, pachygyria) (2) | |  |  | |
| organisational disturbance (dysplasia, schizencephalia, polymicrogyria) (3) | |  |  | |
| other cerebral abnormality, explanatory | | | | |
| **Other specific findings**  none (0) |  | |  |  |
| Chiari malformation (1–5) |  | |  |  |
| Dandy-Walker (6) |  | |  |  |
| dysplasia of the diencephalic-mesencephalic junction (7) |  | |  |  |
| **Bleeds or other insults**  none (0)  explanatory |  | |  |  |
| **Other pathology or significant findings** (explanatory) |  | |  |  |
|  | | | | |

**See Supplementary File 2 (.xlsl) for Suppelementary Table S3: Identified genetic variants.**


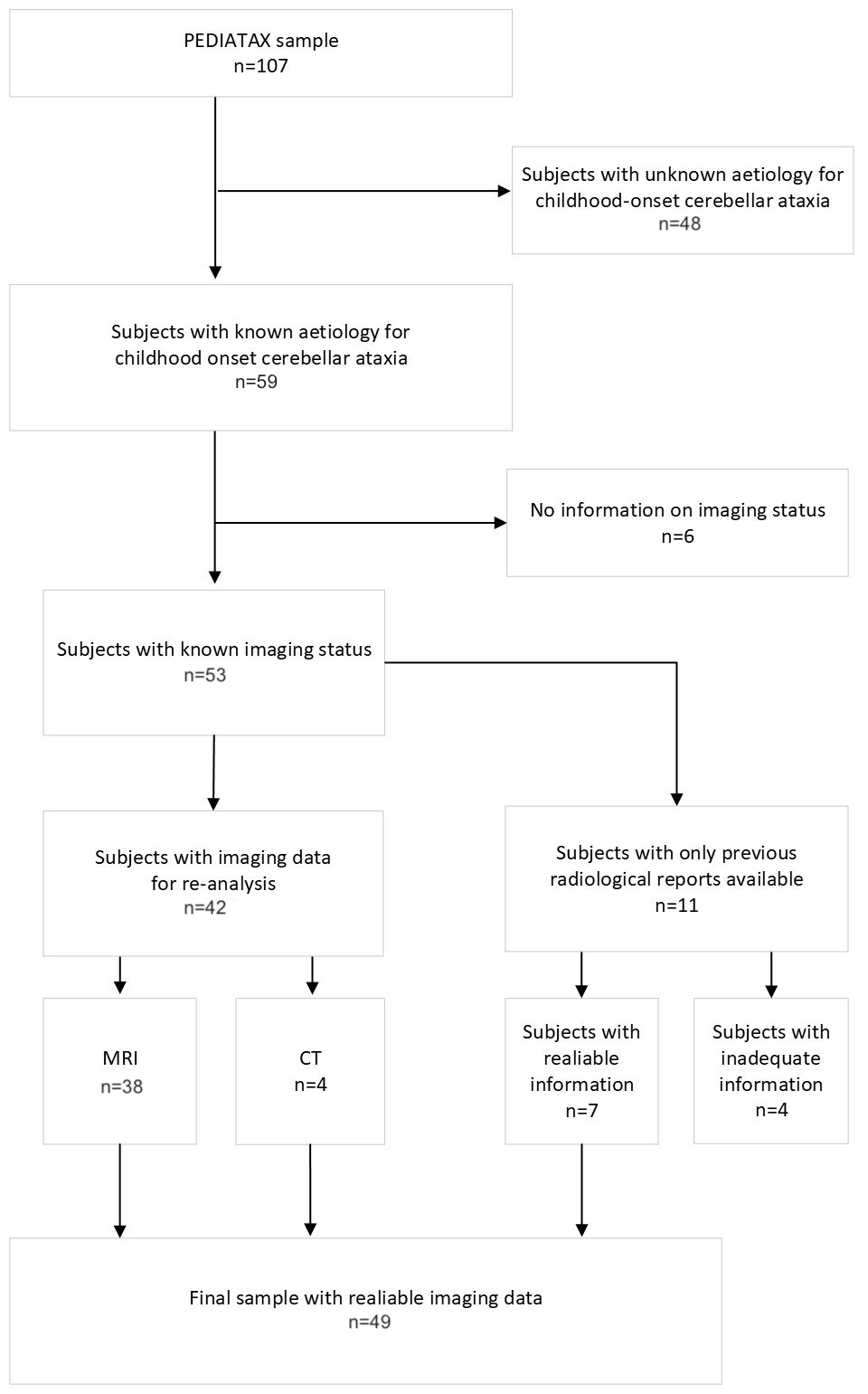


**Supplementary Figure S1.** Imaging data collection.

Abbreviations: CT = computed tomography, MRI = magnetic resonance imaging.
