# Supplementary material for: Phenotypic Heterogeneity in Genetic and Acquired Pediatric Cerebellar Disorders
Source: Mov Disord. 2025 May 6;40(9):1851–62. doi: 10.1002/mds.30210 (PMC12485580; doi:10.1002/mds.30210)
Supplement: Supplementary file 3 — File S3. A list of articles included in the review of the literature. [file MDS-40-1851-s002.docx]

**SF3**. References for the literature cohort.

**Included genes** (corresponding references):

*ANO3* (1–9)

*ATM* (10-62)

*CACNA1A* (63-162)

*CSTB* (163-171)

*ITPR1* (172-178)

*MTM1* (179-222)

*PRRT2* (223-268)

*SIL1* (269-275)

*SLC17A5* (276-292)

*SLC2A1* (293-392)

*SPAST* (393-411)

*TWNK* (412-417)

*BCKDHB* (418-437)

*FXN* (438-465)

*GCH1* (466-515)

*RARS2* (516-531)

*SAMD9L* (532-535)

**References:**

1. Yoo D, Kim HJ, Lee JS, Lee S, Kim SY, Choi M, et al. Early-onset generalized dystonia starting in the lower extremities in a patient with a novel ANO3 variant. Parkinsonism Relat Disord. 2018 May;50:124–5.
2. Nelin S, Hussey R, Faux BM, Rohena L. Youngest presenting patient with dystonia 24 and review of the literature. Clin Case Rep. 2018 Nov;6(11):2070–4.
3. Tunc S, Denecke J, Olschewski L, Bäumer T, Münchau A, Lessel D, et al. A recurrent de-novo ANO3 mutation causes early-onset generalized dystonia. J Neurol Sci. 2019 Jan 15;396:199–201.
4. Kuo MC, Lin HI, Lin CH. Craniocervical dystonia with levodopa-responsive parkinsonism co-segregating with a pathogenic ANO3 mutation in a Taiwanese family. Parkinsonism Relat Disord. 2019 May;62:236–8.
5. Delamarre A, Chelly J, Guehl D, Drouot N, Tranchant C, Anheim M, et al. Novel anoctamin-3 missense mutation responsible for early-onset myoclonic dystonia. Parkinsonism Relat Disord. 2019 Jul;64:346–8.
6. Laurencin C, Broussolle E, Danaila T, Anheim M, Chelly J, Thobois S. A novel heterozygous ANO3 mutation responsible for myoclonic dystonia. J Neurol Sci. 2019 Aug 15;403:65–6.
7. Miocinovic S, Vengoechea J, LeDoux MS, Isbaine F, Jinnah HA. Combined occurrence of deleterious TOR1A and ANO3 variants in isolated generalized dystonia. Parkinsonism Relat Disord. 2020 Apr;73:55–6.
8. Jiménez de Domingo A, Lopez-Martín S, Albert J, Jiménez de la Peña M, Tirado P, Fernández-Mayoralas DM, et al. ANO3 and early-onset dyskinetic encephalopathy. Eur J Med Genet. 2020 Dec;63(12):104085.
9. Carvalho V, Martins J, Correia F, Costa M, Massano J, Temudo T. Another Twist in the Tale: Intrafamilial Phenotypic Heterogeneity in ANO3-Related Dystonia. Mov Disord Clin Pract. 2021 Jul;8(5):758–62.
10. Abolhassani H, Vosughimotlagh A, Asano T, Landegren N, Boisson B, Delavari S, et al. X-Linked TLR7 Deficiency Underlies Critical COVID-19 Pneumonia in a Male Patient with Ataxia-Telangiectasia. J Clin Immunol. 2022 Jan 23;42(1):1–9.
11. Aghamohammadi A, Imai K, Moazzami K, Abolhassani H, Tabatabaeiyan M, Parvaneh N, et al. Ataxia-Telangiectasia in a Patient Presenting With Hyper-immunoglobulin M Syndrome A Aghamohammadi, et al. J Investig Allergol Clin Immunol. 2010;20(5):442–5.
12. Alonazi NA, Hundallah KJ, Al Hashem AM, Mohamed S. A novel variant in ATM gene causes ataxia telangiectasia revealed by whole-exome sequencing. Neurosciences (Riyadh). 2018 Apr 1;23(2):162–4.
13. Amirifar P, Yazdani R, Moeini Shad T, Ghanadan A, Abolhassani H, Lavin M, et al. Cutaneous Granulomatosis and Class Switching Defect as a Presenting Sign in Ataxia-Telangiectasia: First Case from the National Iranian Registry and Review of the Literature. Immunol Invest. 2020 Aug 17;49(6):597–610.
14. Angèle S, Laugé A, Fernet M, Moullan N, Beauvais P, Couturier J, et al. Phenotypic cellular characterization of an ataxia telangiectasia patient carrying a causal homozygous missense mutation. Hum Mutat. 2003;21(2):169–70.
15. Arias DP, Casas AA, Cajal MD, Ramón ML, Montañés LJ. [Complete heart block and asystole in a child with ataxia-telangiectasia]. Arch Argent Pediatr. 2017 Oct 1;115(5):e291–3.
16. Arslan Ateş E, Türkyılmaz A, Eltan SB, Barış S, Güney AI. A Novel ATM Gene Mutation Affecting Splicing in an Ataxia-Telangiectasia Patient. Mol Syndromol. 2022 Feb 1;13(1):80–4.
17. Austen B, Barone G, Reiman A, Byrd PJ, Baker C, Starczynski J, et al. Pathogenic ATM mutations occur rarely in a subset of multiple myeloma patients. Br J Haematol. 2008;142(6):925–33.
18. Bakhtiar S, Woelke S, Huenecke S, Kieslich M, Taylor AM, Schubert R, et al. Pre-emptive Allogeneic Hematopoietic Stem Cell Transplantation in Ataxia Telangiectasia. Front Immunol. 2018 Oct 29;9.
19. Van Belzen MJ, Hiel JAP, Weemaes CMR, Gabreëls FJM, Van Engelen BGM, Smeets DFCM, et al. A double missense mutation in the ATM gene of a Dutch family with ataxia telangiectasia. Hum Genet. 1998;102(2):187–91.
20. Brasseur B, Beauloye V, Chantrain C, Daumerie C, Vermylen C, Waignein F, et al. Papillary thyroid carcinoma in a 9-year-old girl with ataxia-telangiectasia. Pediatr Blood Cancer. 2008 May;50(5):1058–60.
21. Cantarutti N, Claps A, Angelino G, Chessa L, Callea F, El Hachem M, et al. Multi-drugs resistant acne rosacea in a child affected by Ataxia-Telangiectasia: successful treatment with Isotretinoin. Ital J Pediatr. 2015 Mar 28;41(1).
22. Caputi C, Federici G, Soddu S, Travaglini L, Piane M, Bertini E, et al. Mild Neurological Phenotype Associated with Hypomorphic Variants in the Ataxia-Telangiectasia Mutated Gene. Mov Disord Clin Pract. 2022 Jan 1;10(1):124–9.
23. Celiksoy MH, Ozyavuz Cubuk P, Guner SN, Yildiran A. A Case of Ataxia-telangiectasia Presented With Hemophagocytic Syndrome. J Pediatr Hematol Oncol. 2018 Nov 1;40(8):e547–9.
24. Chen W, Liu S, Hu H, Chen G, Zhu S, Jia B, et al. Novel homozygous ataxia‑telangiectasia (A‑T) mutated gene mutation identified in a Chinese pedigree with A‑T. Mol Med Rep. 2019;20(2):1655–62.
25. Czarny J, Andrzejewska M, Zając-Spychała O, Latos-Grażyńska E, Pastorczak A, Wypyszczak K, et al. Successful Treatment of Large B-Cell Lymphoma in a Child with Compound Heterozygous Mutation in the ATM Gene. Int J Mol Sci. 2023 Jan 1;24(2).
26. Danby CS, Allen L, Moharir MD, Weitzman S, Dumont T. Non-hodgkin B-cell lymphoma of the ovary in a child with Ataxia-telangiectasia. J Pediatr Adolesc Gynecol. 2013 Apr;26(2).
27. Fukao T, Tashita H, Teramoto T, Inoue R, Kaneko H, Komiyama K, et al. Novel exonic mutation (5319 G to A) resulting in two aberrantly spliced transcripts of the ATM gene in a Japanese patient with ataxia-telangiectasia. Hum Mutat. 1998;Suppl 1:S223–5.
28. García-Pérez MA, Allende LM, Corell A, Varela P, Moreno AA, Sotoca A, et al. Novel mutations and defective protein kinase C activation of T-lymphocytes in ataxia telangiectasia. Clin Exp Immunol. 2001;123(3):472–80.
29. Heidarzadeh Arani M, ArefNezhad R, Fathgharib J, Aghamohammadi A, Motedayyen H. Clinical complications and their management in a child with ataxia-telangiectasia (A-T): A case report study. Clin Case Rep. 2020 Jan 1;9(1):556–9.
30. Hettiarachchi D, Panchal H, Pathirana BAPS, Rathnayaka PD, Padeniya A, Lai PS, et al. Six Novel ATM Gene Variants in Sri Lankan Patients with Ataxia Telangiectasia. Case Rep Genet. 2020 Dec 9;2020:1–7.
31. Huh HJ, Cho KH, Lee JE, Kwon MJ, Ki CS, Lee PH. Identification of ATM mutations in Korean siblings with ataxia-telangiectasia. Ann Lab Med. 2013;33(3):217–20.
32. Jacobs MF, Robinson D, Wu YM, Opipari VP, Mody R. Homozygous ATM mutation due to germline uniparental isodisomy in patient with T acute lymphoblastic leukemia and hepatosplenic T-cell lymphoma. Cancer Genet. 2022 Aug 1;266–267:15–8.
33. Jeong H, Huh HJ, Youn J, Kim JS, Cho JW, Ki CS. Ataxia-telangiectasia with novel splicing mutations in the ATM gene. Ann Lab Med. 2014;34(1):80–4.
34. Li XL, Wang YL. Ataxia-telangiectasia complicated with Hodgkin’s lymphoma: A case report. World J Clin Cases. 2020 Jun 1;8(11):2387–91.
35. Lnu P, Sehgal V, Kapila S, Gulati N, Bhalla Sehgal L. Ataxia Telangiectasia Presenting as Cervical Dystonia. Cureus. 2022 Oct 26;14(10):e30723.
36. Mandola AB, Reid B, Sirror R, Brager R, Dent P, Chakroborty P, et al. Ataxia Telangiectasia Diagnosed on Newborn Screening-Case Cohort of 5 Years’ Experience. Front Immunol. 2019 Dec 20;10:2940.
37. Maroilley T, Wright NAM, Diao C, MacLaren L, Pfeffer G, Sarna JR, et al. Case Report: Biallelic Loss of Function ATM due to Pathogenic Synonymous and Novel Deep Intronic Variant c.1803-270T > G Identified by Genome Sequencing in a Child With Ataxia-Telangiectasia. Front Genet. 2022 Jan 25;13.
38. Martin-Rodriguez S, Calvo-Ferrer A, Ortega-Unanue N, Samaniego-Jimenez L, Sanz-Izquierdo MP, Bernardo-Gonzalez I. Two novel variants in the ATM gene causing ataxia-telangiectasia, including a duplication of 90 kb: Utility of targeted next-generation sequencing in detection of copy number variation. Ann Hum Genet. 2019 Jul 1;83(4):266–73.
39. Miasaki FY, Saito KC, Yamamoto GL, Boguszewski CL, De Carvalho GA, Kimura ET, et al. Thyroid and Breast Cancer in 2 Sisters With Monoallelic Mutations in the Ataxia Telangiectasia Mutated ( ATM) Gene. J Endocr Soc. 2022 Apr 1;6(4):1–4.
40. Mijalovsky A, Halperin D, Perez Y, Zafarov B, Shaco-Levy R, Kapelushnik J, et al. Malignant Peritoneal Mesothelioma in an Infant With Familial ATM Mutations. J Pediatr Hematol Oncol. 2018 Nov 1;40(8):e511–5.
41. Minto H, Mensah KA, Reynolds PR, Meffre E, Rubtsova K, Gelfand EW. A novel ATM mutation associated with elevated atypical lymphocyte populations, hyper-IgM, and cutaneous granulomas. Clin Immunol. 2019 Mar 1;200:55–63.
42. Mortaz E, Marashian SM, Ghaffaripour H, Varahram M, Mehrian P, Dorudinia A, et al. A new ataxia-telangiectasia mutation in an 11-year-old female. Immunogenetics. 2017 Jul 1;69(7):415–9.
43. Nakayama T, Sato Y, Uematsu M, Takagi M, Hasegawa S, Kumada S, et al. Myoclonic axial jerks for diagnosing atypical evolution of ataxia telangiectasia. Brain Dev. 2015 Mar 1;37(3):362–5.
44. Necpál J, Zech M, Škorvánek M, Havránková P, Fečíková A, Winkelmann J, et al. Ataxia Telangiectasia Gene Mutation in Isolated Segmental Dystonia Without Ataxia and Telangiectasia. Mov Disord Clin Pract. 2017 Jan 1;5(1):89–91.
45. Perreault S, Bernard G, Lortie A, Le Deist F, Decaluwe H. Ataxia-telangiectasia presenting with a novel immunodeficiency. Pediatr Neurol. 2012 May;46(5):322–4.
46. Piane M, Molinaro A, Soresina A, Costa S, Maffeis M, Germani A, et al. Novel compound heterozygous mutations in a child with Ataxia-Telangiectasia showing unrelated cerebellar disorders. J Neurol Sci. 2016 Dec 15;371:48–53.
47. Pietrucha BM, Heropolitańska-Pliszka E, Wakulińska A, Skopczyńska H, Gatti RA, Bernatowska E. Ataxia-telangiectasia with hyper-IgM and Wilms tumor: fatal reaction to irradiation. J Pediatr Hematol Oncol. 2010;32(1):e28–30.
48. Roohi J, Crowe J, Loredan D, Anyane-Yeboa K, Mansukhani MM, Omesi L, et al. New diagnosis of atypical ataxia-telangiectasia in a 17-year-old boy with T-cell acute lymphoblastic leukemia and a novel ATM mutation. J Hum Genet. 2017 Apr 1;62(5):581–4.
49. Ruiz-Botero F, Rodríguez-Guerrero JT. [New mutation in ATM gen in patient whith Ataxia Telangiectasia: Clinical case]. Rev Chil Pediatr. 2017 Jul 1;88(4):524–8.
50. Schröder S, Wieland B, Ohlenbusch A, Yigit G, Altmüller J, Boltshauser E, et al. Evidence of pathogenicity for the leaky splice variant c.1066-6T>G in ATM. Am J Med Genet A. 2020 Dec 1;182(12):2971–5.
51. Shalash AS, Rösler TW, Salama M, Pendziwiat M, Müller SH, Hopfner F, et al. Evidence for pathogenicity of variant ATM Val1729Leu in a family with ataxia telangiectasia. Neurogenetics. 2021 May 1;22(2):143–7.
52. Sharapova SO, Valochnik A V., Guryanova IE, Sakovich IS, Aleinikova O V. Novel biallelic ATM mutations coexist with a mosaic form of triple X syndrome in an 11-year-old girl at remission after T cell acute leukemia. Immunogenetics. 2018 Sep 1;70(9):613–7.
53. Sherkat R, Moghaddam NA, Reisi N, Rezaei M. Spontaneous Regression of Diffuse Large B-cell Lymphoma in a Patient with Ataxia-Telangiectasia. Adv Biomed Res. 2022 Jan 1;11(1):31.
54. Silvestri G, Masciullo M, Piane M, Savio C, Modoni A, Santoro M, et al. Homozygosity for c 6325T>G transition in the ATM gene causes an atypical, late-onset variant form of ataxia-telangiectasia. J Neurol. 2010 Oct;257(10):1738–40.
55. Soresina A, Meini A, Lougaris V, Cattaneo G, Pellegrino S, Piane M, et al. Different clinical and immunological presentation of ataxia-telangiectasia within the same family. Neuropediatrics. 2008 Feb;39(1):43–5.
56. Tabatabaiefar MA, Alipour P, Pourahmadiyan A, Fattahi N, Shariati L, Golchin N, et al. A novel pathogenic variant in an Iranian Ataxia telangiectasia family revealed by next-generation sequencing followed by in silico analysis. J Neurol Sci. 2017 Aug 15;379:212–6.
57. Tangsinmankong N, Wayne AS, Howenstine MS, Washington KR, Langston C, Gatti RA, et al. Lymphocytic interstitial pneumonitis, elevated IgM concentration, and hepatosplenomegaly in ataxia-telangiectasia. J Pediatr. 2001;138(6):939–41.
58. Toyoshima M, Hara T, Zhang H, Yamamoto T, Akaboshi S, Nanba E, et al. Ataxia-Telangiectasia Without Immunodeficiency: Novel Point Mutations Within and Adjacent to the Phosphatidylinositol 3-Kinase-Like Domain. J Med Genet. 1998;75:141–4.
59. Ulusoy E, Edeer-Karaca N, Özen S, Ertan Y, Gökşen D, Aksu G, et al. An unusual manifestation: Papillary thyroid carcinoma in a patient with ataxia-telengiectasia. Turk J Pediatr. 2016;58(4):442–5.
60. Worth PF, Srinivasan V, Smith A, Last JI, Wootton LL, Biggs PM, et al. Very mild presentation in adult with classical cellular phenotype of ataxia telangiectasia. Mov Disord. 2013 Apr;28(4):524–8.
61. Zaki-Dizaji M, Tajdini M, Kiaee F, Shojaaldini H, Badv RS, Abolhassani H, et al. Dystonia in Ataxia Telangiectasia: A Case Report with Novel Mutations. Oman Med J. 2020;35(1).
62. Zhang L, Jia Y, Qi X, Li M, Wang S, Wang Y. Trihexyphenidyl for treatment of dystonia in ataxia telangiectasia: a case report. Childs Nerv Syst. 2020 Apr 1;36(4):873–5.
63. Algahtani H, Shirah B, Algahtani R, Al-Qahtani MH, Abdulkareem AA, Naseer MI. A novel mutation in CACNA1A gene in a Saudi female with episodic ataxia type 2 with no response to acetazolamide or 4-aminopyridine. Intractable Rare Dis Res. 2019;8(1):67–71.
64. Alonso I, Barros J, Tuna A, Seixas A, Coutinho P, Sequeiros J, et al. A novel R1347Q mutation in the predicted voltage sensor segment of the P/Q-type calcium-channel alpha-subunit in a family with progressive cerebellar ataxia and hemiplegic migraine. Clin Genet. 2004 Jan;65(1):70–2.
65. Angelini C, Van Gils J, Bigourdan A, Jouk PS, Lacombe D, Menegon P, et al. Major intra-familial phenotypic heterogeneity and incomplete penetrance due to a CACNA1A pathogenic variant. Eur J Med Genet. 2019 Jun 1;62(6).
66. Arteche-López A, Álvarez-Mora M, Sánchez Calvin M, Lezana Rosales J, Palma Milla C, Gómez Rodríguez MJ, et al. Biallelic variants in genes previously associated with dominant inheritance: CACNA1A, RET and SLC20A2. Eur J Hum Genet. 2021 Oct 1;29(10):1520–6.
67. Balck A, Tunc S, Schmitz J, Hollstein R, Kaiser FJ, Brüggemann N. A Novel Frameshift CACNA1A Mutation Causing Episodic Ataxia Type 2. Cerebellum. 2018 Aug 1;17(4):504–6.
68. Batum M, Kısabay Ak A, Çetin G, Çelebi HBG, Çam S, Mavioğlu H. Coincidental occurance of episodic ataxia and multiple sclerosis: a case report and review of the literature. Int J Neurosci. 2022;132(7):656–61.
69. Bertholon P, Chabrier S, Riant F, Tournier-Lasserve E, Peyron R. Episodic ataxia type 2: unusual aspects in clinical and genetic presentation. Special emphasis in childhood. J Neurol Neurosurg Psychiatry. 2009;80(11):1289–92.
70. Blumkin L, Michelson M, Leshinsky-Silver E, Kivity S, Lev D, Lerman-Sagie T. Congenital ataxia, mental retardation, and dyskinesia associated with a novel CACNA1A mutation. J Child Neurol. 2010;25(7):892–7.
71. Bolte KN, Assaf M, Zach T, Peche S. Two Children with Early-Onset Strokes and Intractable Epilepsy, Both with CACNA1A Mutations. Child Neurol Open. 2022 Jan;9:1–7.
72. Bruun M, Hjermind LE, Thomsen C, Danielsen E, Thomsen LL, Pinborg LH, et al. Familial hemiplegic migraine type 1 associated with parkinsonism: a case report. Case Rep Neurol. 2015 May 22;7(1):84–9.
73. Byers HM, Beatty CW, Hahn SH, Gospe SM. Dramatic Response After Lamotrigine in a Patient With Epileptic Encephalopathy and a De Novo CACNA1A Variant. Pediatr Neurol. 2016 Jul 1;60:79–82.
74. Camia F, Pisciotta L, Morana G, Schiaffino MC, Renna S, Carrera P, et al. Combined early treatment in hemiplegic attacks related to CACNA1A encephalopathy with brain oedema: Blocking the cascade? Cephalalgia. 2017 Oct 1;37(12):1202–6.
75. Carreño O, García-Silva MT, García-Campos A, Martínez-De Aragõn A, Cormand B, MacAya A. Acute striatal necrosis in hemiplegic migraine with de novo CACNA1A mutation. Headache. 2011 Nov;51(10):1542–6.
76. Chang MY, Yuen T, Vyas A, Borchert MS. Paroxysmal tonic upgaze (PTU) associated with CACNA1A mutation and gross motor delay. J AAPOS. 2021 Dec 1;25(6):353-353.e1.
77. Cleves C, Parikh S, Rothner AD, Tepper SJ. Link between confusional migraine, hemiplegic migraine and episodic ataxia type 2: hypothesis, family genealogy, gene typing and classification. Cephalalgia. 2010 Jun;30(6):740–3.
78. Cricchi F, Di Lorenzo C, Grieco GS, Rengo C, Cardinale A, Racaniello M, et al. Early-onset progressive ataxia associated with the first CACNA1A mutation identified within the I-II loop. J Neurol Sci. 2007 Mar 15;254(1–2):69–71.
79. Curtain RP, Smith RL, Ovcaric M, Griffiths LR. Minor head trauma-induced sporadic hemiplegic migraine coma. Pediatr Neurol. 2006 Apr;34(4):329–32.
80. Debiais S, Hommet C, Bonnaud I, Barthez MA, Rimbaux S, Riant F, et al. The FHM1 mutation S218L: a severe clinical phenotype? A case report and review of the literature. Cephalalgia. 2009 Dec;29(12):1337–9.
81. Du X, Chen Y, Zhao Y, Luo W, Cen Z, Hao W. Dramatic response to pyridoxine in a girl with absence epilepsy with ataxia caused by a de novo CACNA1A mutation. Seizure. 2017 Feb 1;45:189–91.
82. Dziewulska D, Kierdaszuk B. Ultrastructural changes in microvessels in familial hemiplegic migraine with CACNA1A mutation. Clin Neuropathol. 2018 Nov 1;37(6):283–7.
83. Epperson M V., Haws ME, Standridge SM, Gilbert DL. An Atypical Rett Syndrome Phenotype Due to a Novel Missense Mutation in CACNA1A. J Child Neurol. 2018 Mar 1;33(4):286–9.
84. Fujioka S, Rayaprolu S, Sundal C, Broderick DF, Langley WA, Shoffner J, et al. A novel de novo pathogenic mutation in the CACNA1A gene. Mov Disord. 2012 Oct;27(12):1578–9.
85. Gandini MA, Souza IA, Ferron L, Innes AM, Zamponi GW. The de novo CACNA1A pathogenic variant Y1384C associated with hemiplegic migraine, early onset cerebellar atrophy and developmental delay leads to a loss of Cav2.1 channel function. Mol Brain. 2021 Dec 1;14(1).
86. García Segarra N, Gautschi I, Mittaz-Crettol L, Kallay Zetchi C, Al-Qusairi L, Van Bemmelen MX, et al. Congenital ataxia and hemiplegic migraine with cerebral edema associated with a novel gain of function mutation in the calcium channel CACNA1A. J Neurol Sci. 2014 Jul;342(1–2):69–78.
87. García-Baró-Huarte M, Iglesias-Mohedano AM, Slöcker-Barrio M, Vázquez-López M, García-Morín M, Miranda-Herrero MC, et al. Phenotypic variability in a four generation family with a p.Thr666Met CACNA1A gene mutation. Pediatr Neurol. 2014 Oct 1;51(4):557–9.
88. Gauquelin L, Hawkins C, Tam EWY, Miller SP, Yoon G. Pearls & Oy-sters: Fatal brain edema is a rare complication of severe CACNA1A-related disorder. Neurology. 2020 Apr 7;94(14):631–4.
89. Geerlings RP, Koehler PJ, Haane DY, Stam AH, De Vries B, Boon EM, et al. Head tremor related to CACNA1A mutations. Cephalalgia. 2011 Sep;31(12):1315–9.
90. Giffinmrcp NJ, Benton S, Goadsby PJ. Benign paroxysmal torticollis of infancy: four new cases and linkage to CACNA1A mutation. Dev Med Child Neurol. 2002;44(7):490–3.
91. González-Mingot C, López-Ortega R, Brieva-Ruiz L. The efficacy of combining topiramate and 4-aminopyridine to reduce relapses and interictal progression in two cases of episodic ataxia type 2. Neurol Sci. 2022 Aug 1;43(8):5099–101.
92. Gudenkauf FJ, Azamian MS, Hunter J V., Nayak A, Lalani SR. A novel CACNA1A variant in a child with early stroke and intractable epilepsy. Mol Genet Genomic Med. 2020 Oct 1;8(10).
93. Guerin AA, Feigenbaum A, Donner EJ, Yoon G. Stepwise developmental regression associated with novel CACNA1A mutation. Pediatr Neurol. 2008 Nov;39(5):363–4.
94. Guida S, Trettel F, Pagnutti S, Mantuano E, Tottene A, Veneziano L, et al. Complete loss of P/Q calcium channel activity caused by a CACNA1A missense mutation carried by patients with episodic ataxia type 2. Am J Hum Genet. 2001;68(3):759–64.
95. Guterman EL, Yurgionas B, Nelson AB. Pearls & Oy-sters: Episodic ataxia type 2: Case report and review of the literature. Neurology. 2016 Jun 6;86(23):e239.
96. Harno H, Hirvonen T, Kaunisto MA, Aalto H, Levo H, Isotalo E, et al. Acetazolamide improves neurotological abnormalities in a family with episodic ataxia type 2 (EA-2). J Neurol. 2004 Feb;251(2):232–4.
97. Harries AM, Sandhu M, Spacey SD, Aly MM, Honey CR. Unilateral pallidal deep brain stimulation in a patient with dystonia secondary to episodic ataxia type 2. Stereotact Funct Neurosurg. 2013 Jun;91(4):233–5.
98. Hayashida T, Saito Y, Ishii A, Yamada H, Itakura A, Minato T, et al. CACNA1A-related early-onset encephalopathy with myoclonic epilepsy: A case report. Brain Dev. 2018 Feb 1;40(2):130–3.
99. Hirasawa-Inoue A, Ishiyama A, Takeshita E, Shimizu-Motohashi Y, Saito T, Komaki H, et al. Single-fiber electromyography-based diagnosis of CACNA1A mutation in children: A potential role of the electrodiagnosis in the era of whole exome sequencing. Brain Dev. 2019 Nov 1;41(10):905–9.
100. Ho CY, Love HL, Sokol DK, Walsh LE. Longitudinal MRI brain findings in the R1349Q pathogenic variant of CACNA1A. Radiol Case Rep. 2021 Jun 1;16(6):1276–9.
101. Hu Y, Jiang H, Wang Q, Xie Z, Pan S. Identification of a novel nonsense mutation p.Tyr1957Ter of CACNA1A in a Chinese family with episodic ataxia 2. PLoS One. 2013 Feb 18;8(2).
102. Idiculla PS, Siddiqui JH. A case of novel CACNA1A mutation causing type 2 episodic ataxia. Neurol Sci. 2021 Jun 1;42(6):2577–8.
103. Indelicato E, Nachbauer W, Eigentler A, Donnemiller E, Wagner M, Unterberger I, et al. Ten years of follow-up in a large family with familial hemiplegic migraine type 1: Clinical course and implications for treatment. Cephalalgia. 2018 May 1;38(6):1167–76.
104. Isaacs DA, Bradshaw MJ, Brown K, Hedera P. Case report of novel CACNA1A gene mutation causing episodic ataxia type 2. SAGE Open Med Case Rep. 2017 Jan 1;5:1–3.
105. Ji XN, Xu CJ, Gao ZJ, Chen SH, Xu KM, Chen Q. [Glucose transporter 1 deficiency syndrome: features of movement disorders, diagnosis and treatment]. Zhongguo Dang Dai Er Ke Za Zhi. 2018 Mar 1;20(3):209–13.
106. Jouvenceau A, Eunson LH, Spauschus A, Ramesh V, Zuberi SM, Kullmann DM, et al. Human epilepsy associated with dysfunction of the brain P/Q-type calcium channel. Lancet. 2001 Sep 8;358(9284):801–7.
107. Kaido M, Furuta M, Nakamori M, Yuasa Y, Takahashi MP. [Episodic ataxia type 2 manifests as epileptiform electroencephalographic activity with no epileptic attacks in two family members]. Rinsho Shinkeigaku. 2016;56(4):260–4.
108. Kim HJ, Kim JS, Choi JH, Shin JH, Choi KD, Zee DS. Rebound upbeat nystagmus after lateral gaze in episodic ataxia type 2. Cerebellum. 2014;13(3):411–3.
109. Kinder S, Ossig C, Wienecke M, Beyer A, Von Der Hagen M, Storch A, et al. Novel frameshift mutation in the CACNA1A gene causing a mixed phenotype of episodic ataxia and familiar hemiplegic migraine. Eur J Paediatr Neurol. 2015 Jan 1;19(1):72–4.
110. Knierim E, Leisle L, Wagner C, Weschke B, Lucke B, Bohner G, et al. Recurrent stroke due to a novel voltage sensor mutation in Cav2.1 responds to verapamil. Stroke. 2011 Feb;42(2).
111. Kors EE, Melberg A, Vanmolkot KRJ, Kumlien E, Haan J, Raininko R, et al. Childhood epilepsy, familial hemiplegic migraine, cerebellar ataxia, and a new CACNA1A mutation. Neurology. 2004 Sep 29;63(6):1136–7.
112. Kubota T, Nabatame S, Sato R, Hama M, Nishiike U, Mochizuki H, et al. Hemiplegic migraine type 2 caused by a novel variant within the P-type ATPase motif in ATP1A2 concomitant with a CACNA1A variant. Brain Dev. 2021 Oct 1;43(9):952–7.
113. Lance S, Mossman S, Poke G. A Novel CACNA1A Nonsense Variant [c.4054C>T (p.Arg1352⁎)] Causing Episodic Ataxia Type 2. Case Rep Neurol Med. 2018;2018:1–3.
114. Li M, Zheng X, Zhong R, Zhao Q, Lu Y, Wang Z, et al. Familial Hemiplegic Migraine With Progressive Cerebellar Ataxia Caused by a p.Thr666Met CACNA1A Gene Mutation in a Chinese Family. Front Neurol. 2019 Nov 19;10:1221.
115. Luan H, Zhang L, Zhang S, Zhang M. Next-generation sequencing identified a novel CACNA1A I1379F variant in a familial hemiplegic migraine type 1 pedigree: A case report. Medicine (Baltimore). 2021 Dec 23;100(51):e28141.
116. Luo X, Rosenfeld JA, Yamamoto S, Harel T, Zuo Z, Hall M, et al. Clinically severe CACNA1A alleles affect synaptic function and neurodegeneration differentially. PLoS Genet. 2017 Jul 1;13(7).
117. Magis D, Boon E, Coppola G, Daron A, Schoenen J. A novel CACNA1A mutation results in episodic ataxia with migrainous features without headache. Cephalalgia. 2012 Nov;32(15):1147–9.
118. Malamud E, Otallah SI. Use of Dalfampridine in a Young Child with Episodic Ataxia Type 2. Child Neurol Open. 2022 Jan;9:1–3.
119. Malpas TJ, Riant F, Tournier-Lasserve E, Vahedi K, Neville BGR. Sporadic hemiplegic migraine and delayed cerebral oedema after minor head trauma: a novel de novo CACNA1A gene mutation. Dev Med Child Neurol. 2010;52(1):103–4.
120. Martínez‐monseny AF, Edo A, Casas‐alba D, Izquierdo‐serra M, Bolasell M, Conejo D, et al. CACNA1A Mutations Causing Early Onset Ataxia: Profiling Clinical, Dysmorphic and Structural-Functional Findings. Int J Mol Sci. 2021 May 2;22(10):2–14.
121. Matsuyama Z, Murase M, Shimizu H, Aoki Y, Hayashi M, Hozumi I, et al. A novel insertion mutation of acetazolamide-responsive episodic ataxia in a Japanese family. J Neurol Sci. 2003 Jun 15;210(1–2):91–3.
122. Myers CT, McMahon JM, Schneider AL, Petrovski S, Allen AS, Carvill GL, et al. De Novo Mutations in SLC1A2 and CACNA1A Are Important Causes of Epileptic Encephalopathies. Am J Hum Genet. 2016 Aug 4;99(2):287–98.
123. Na S, Kim T. Efficacy of levetiracetam in patients with episodic ataxia type 2 caused by CACNA1A mutation: three case reports. Neurol Sci. 2021 Sep 1;42(9):3897–9.
124. Nachbauer W, Nocker M, Karner E, Stankovic I, Unterberger I, Eigentler A, et al. Episodic ataxia type 2: phenotype characteristics of a novel CACNA1A mutation and review of the literature. J Neurol. 2014;261(5):983–91.
125. Naik S, Pohl K, Malik M, Siddiqui A, Josifova D. Early-onset cerebellar atrophy associated with mutation in the CACNA1A gene. Pediatr Neurol. 2011 Nov;45(5):328–30.
126. Nardello R, Plicato G, Mangano GD, Gennaro E, Mangano S, Brighina F, et al. Two distinct phenotypes, hemiplegic migraine and episodic Ataxia type 2, caused by a novel common CACNA1A variant. BMC Neurol. 2020 Apr 26;20(1).
127. Ogawa Y, Nakamura K, Ezawa N, Yamaguchi T, Yoshinaga T, Miyazaki D, et al. A novel CACNA1A nonsense variant in a patient presenting with paroxysmal exertion-induced dyskinesia. J Neurol Sci. 2019 Apr 15;399:214–6.
128. Ohmura K, Suzuki Y, Saito Y, Wada T, Goto M, Seto S. Sporadic hemiplegic migraine presenting as acute encephalopathy. Brain Dev. 2012 Sep;34(8):691–5.
129. Omata T, Takanashi J ichi, Wada T, Arai H, Tanabe Y. Genetic diagnosis and acetazolamide treatment of familial hemiplegic migraine. Brain Dev. 2011 Apr 1;33(4):332–4.
130. Park D, Kim SH, Lee YJ, Song GJ, Park JS. A novel CACNA1A mutation associated with episodic ataxia 2 presenting with periodic paralysis. Acta Neurol Belg. 2018 Mar 1;118(1):137–9.
131. Pelzer N, Hoogeveen ES, Ferrari MD, Poll-The BT, Kruit MC, Terwindt GM. Brain atrophy following hemiplegic migraine attacks. Cephalalgia. 2018 May 1;38(6):1199–202.
132. Petrovicova A, Brozman M, Kurca E, Gobo T, Dluha J, Kalmarova K, et al. Novel missense variant of CACNA1A gene in a Slovak family with episodic ataxia type 2. Biomed Pap Med Fac Univ Palacky Olomouc Czech Repub. 2017;161(1):107–10.
133. Pradotto L, Mencarelli M, Bigoni M, Milesi A, Di Blasio A, Mauro A. Episodic ataxia and SCA6 within the same family due to the D302N CACNA1A gene mutation. J Neurol Sci. 2016 Dec 15;371:81–4.
134. Reinson K, Õiglane-Shlik E, Talvik I, Vaher U, Õunapuu A, Ennok M, et al. Biallelic CACNA1A mutations cause early onset epileptic encephalopathy with progressive cerebral, cerebellar, and optic nerve atrophy. Am J Med Genet A. 2016 Aug 1;170(8):2173–6.
135. Robbins MS, Lipton RB, Laureta EC, Grosberg BM. CACNA1A nonsense mutation is associated with basilar-type migraine and episodic ataxia type 2. Headache. 2009 Jul;49(7):1042–6.
136. Romaniello R, Zucca C, Tonelli A, Bonato S, Baschirotto C, Zanotta N, et al. A wide spectrum of clinical, neurophysiological and neuroradiological abnormalities in a family with a novel CACNA1A mutation. J Neurol Neurosurg Psychiatry. 2010;81(8):840–3.
137. Sánchez-Albisua I, Schöning M, Jurkat-Rott K, Lerche H. Possible effect of corticoids on hemiplegic attacks in severe hemiplegic migraine. Pediatr Neurol. 2013 Oct;49(4):286–8.
138. Schaare D, Sarasua SM, Lusk L, Parthasarathy S, Wang L, Helbig I, et al. Concomitant Calcium Channelopathies Involving CACNA1A and CACNA1F: A Case Report and Review of the Literature. Genes (Basel). 2023 Feb 1;14(2).
139. Scoggan KA, Chandra T, Nelson R, Hahn AF, Bulman DE. Identification of two novel mutations in the CACNA1A gene responsible for episodic ataxia type 2. J Med Genet. 2001;38(4):249–53.
140. Scoggan KA, Friedman JH, Bulman DE. CACNA1A mutation in a EA-2 patient responsive to acetazolamide and valproic acid. Can J Neurol Sci. 2006 Feb;33(1):68–72.
141. Scoppola C, Magli G, Conti M, Fadda M, Luzzu GM, Simula DM, et al. CACNA1A-Linked Hemiplegic Migraine in GLUT 1 Deficiency Syndrome: A Case Report. Front Neurol. 2021 May 31;12.
142. Shimmura M, Uehara T, Yamashita K, Shigeto H, Yamasaki R, Ishikawa K, et al. Slowed abduction during smooth pursuit eye movement in episodic ataxia type 2 with a novel CACNA1A mutation. J Neurol Sci. 2017 Oct 15;381:4–6.
143. Sivák Š, Kurča E, Krajčiová A, Hikkelová M, Šimko J, Mišovicová N, et al. Novel missense variant of CACNA1A gene: A case report of a family with episodic ataxia type 2. J Neurol Sci. 2017 May 15;376:119–20.
144. Spacey SD, Materek LA, Blajez ;, Szczygielski I, Bird TD. Two Novel CACNA1A Gene Mutations Associated With Episodic Ataxia Type 2 and Interictal Dystonia. Arch Neurol. 2005;62(2):314–6.
145. Spacey SD, Materek LA, Szczygielski BI, Bird TD. Two novel CACNA1A gene mutations associated with episodic ataxia type 2 and interictal dystonia. Arch Neurol. 2005 Feb;62(2):314–6.
146. Sprouse Blum AS, Couperus CJ, Rosen BJ, Haskin-Leahy LF, Shapiro RE. Familial “Diplegic” Migraine - Description of a Family With a Novel CACNA1A Mutation. Headache. 2020 Mar 1;60(3):600–6.
147. Stam AH, Luijckx GJ, Poll-Thé BT, Ginjaar IB, Frants RR, Haan J, et al. Early seizures and cerebral oedema after trivial head trauma associated with the CACNA1A S218L mutation. J Neurol Neurosurg Psychiatry. 2009;80(10):1125–9.
148. Stampfl B, Fee D. Novel Mutation in CACNA1A Associated with Activity-Induced Dystonia, Cervical Dystonia, and Mild Ataxia. Case Rep Neurol Med. 2021 Aug 2;2021:1–4.
149. Stendel C, D’Adamo MC, Wiessner M, Dusl M, Cenciarini M, Belia S, et al. Association of A Novel Splice Site Mutation in P/Q-Type Calcium Channels with Childhood Epilepsy and Late-Onset Slowly Progressive Non-Episodic Cerebellar Ataxia. Int J Mol Sci. 2020 Jun 1;21(11).
150. Stubberud A, O’Connor E, Tronvik E, Houlden H, Matharu M. R1352Q CACNA1A Variant in a Patient with Sporadic Hemiplegic Migraine, Ataxia, Seizures and Cerebral Oedema: A Case Report. Case Rep Neurol. 2021 Jan 1;13(1):123–30.
151. Vila-Pueyo M, Gené GG, Flotats-Bastardes M, Elorza X, Sintas C, Valverde MA, et al. A loss-of-function CACNA1A mutation causing benign paroxysmal torticollis of infancy. Eur J Paediatr Neurol. 2014;18(3):430–3.
152. Vithayathil J, Freeman C, Jacobwitz M, Schwartz ES, Agarwal S. Prolonged neurologic deficits with brain MRI changes following ECT in an adolescent with a CACNA1a-related disorder; a case report. BMC Neurol. 2022 Dec 1;22(1).
153. De Vries B, Stam AH, Beker F, Van Maagdenberg A Den, Vanmolkot KRJ, Laan L, et al. CACNA1A mutation linking hemiplegic migraine and alternating hemiplegia of childhood. Cephalalgia. 2008 Aug;28(8):887–91.
154. Wada T, Kobayashi N, Takahashi Y, Aoki T, Watanabe T, Saitoh S. Wide clinical variability in a family with a CACNA1A T666M mutation: Hemiplegic migraine, coma, and progressive ataxia. Pediatr Neurol. 2002;26(1):47–50.
155. Weyhrauch DL, Ye D, Boczek NJ, Tester DJ, Gavrilova RH, Patterson MC, et al. Whole Exome Sequencing and Heterologous Cellular Electrophysiology Studies Elucidate a Novel Loss-of-Function Mutation in the CACNA1A-Encoded Neuronal P/Q-Type Calcium Channel in a Child With Congenital Hypotonia and Developmental Delay. Pediatr Neurol. 2016 Feb 1;55:46–51.
156. Wong-Spracklen VMY, Kolesnik A, Eck J, Sabanathan S, Spasic-Boskovic O, Maw A, et al. Biallelic CACNA1A variants: Review of literature and report of a child with drug-resistant epilepsy and developmental delay. Am J Med Genet A. 2022 Nov 1;188(11):3306–11.
157. Wu HJ, Lau WL, Chan TYC, Chen SPL, Ko CH. Differentiating episodic ataxia type 2 from migraine: a case report. Hong Kong Med J. 2020;26(6):526–7.
158. Xu Y, Wang Z, Sun Q, Zhou L, Xu H, Hu Y. Clinical features and CACNA1A gene mutation in a family with episodic ataxia type 2. Zhong Nan Da Xue Xue Bao Yi Xue Ban. 2022 Jun 28;47(6):801–8.
159. Yamazaki S, Ikeno K, Abe T, Tohyama J, Adachi Y. Hemiconvulsion-hemiplegia-epilepsy syndrome associated with CACNA1A S218L mutation. Pediatr Neurol. 2011 Sep;45(3):193–6.
160. Yuan X, Zheng Y, Gao F, Sun W, Wang Z, Zhao G. Case Report: A Novel CACNA1A Mutation Caused Flunarizine-Responsive Type 2 Episodic Ataxia and Hemiplegic Migraine With Abnormal MRI of Cerebral White Matter. Front Neurol. 2022 May 23;13.
161. Yue Q, Jen J, Thwe M, Nelson S, Baloh RW. De novo mutation in CACNA1A caused acetazolamide-responsive episodic ataxia. Am J Med Genet. 1998;77(4):298–301.
162. Zafeiriou DI, Lehmann-Horn F, Vargiami E, Teflioudi E, Ververi A, Jurkat-Rott K. Episodic ataxia type 2 showing ictal hyperhidrosis with hypothermia and interictal chronic diarrhea due to a novel CACNA1A mutation. Eur J Paediatr Neurol. 2009 Mar;13(2):191–3.
163. Nokelainen P, Heiskala H, Lehesjoki AE, Kaski M. A patient with 2 different repeat expansion mutations. Arch Neurol. 2000 Aug;57(8):1199–203.
164. Kagitani-Shimono K, Imai K, Okamoto N, Ono J, Okada S. Unverricht-Lundborg disease with cystatin B gene abnormalities. Pediatr Neurol. 2002 Jan;26(1):55–60.
165. Kobayashi K, Matsumoto R, Kondo T, Kawamata J, Hitomi T, Inouchi M, et al. Decreased cortical excitability in Unverricht-Lundborg disease in the long-term follow-up: a consecutive SEP study. Clin Neurophysiol. 2011 Aug;122(8):1617–21.
166. Pinto E, Freitas J, Duarte AJ, Ribeiro I, Ribeiro D, Lima JL, et al. Unverricht-Lundborg disease: homozygosity for a new splicing mutation in the cystatin B gene. Epilepsy Res. 2012 Mar;99(1–2):187–90.
167. Saadah M, El Beshari M, Saadah L, Hamdallah H, Alloub Z, Al Zaabi AA, et al. Progressive myoclonic epilepsy type 1: Report of an Emirati family and literature review. Epilepsy Behav Case Rep. 2014;2:112–7.
168. Assenza G, Benvenga A, Gennaro E, Tombini M, Campana C, Assenza F, et al. A novel c132-134del mutation in Unverricht-Lundborg disease and the review of literature of heterozygous compound patients. Epilepsia. 2017 Feb;58(2):e31–5.
169. O’Brien A, Marshall CR, Blaser S, Ray PN, Yoon G. Severe neurodegeneration, progressive cerebral volume loss and diffuse hypomyelination associated with a homozygous frameshift mutation in CSTB. Eur J Hum Genet. 2017 Jun;25(6):775–8.
170. Mohamadpour M, Gabriel G, Grant AC. A Native Haitian Woman with Unverricht-Lundborg Disease. Case Rep Neurol. 2017;9(3):284–8.
171. Kim KH, Song JS, Park CW, Ki CS, Heo K. First Molecular Diagnosis of a Patient with Unverricht-Lundborg Disease in Korea. Yonsei Med J. 2018 Aug;59(6):798–800.
172. Ngo KJ, Poke G, Neas K, Fogel BL. Spinocerebellar Ataxia type 29 in a family of Māori descent. Cerebellum Ataxias. 2019;6:14.
173. Zachou A, Palaiologou D, Kanavakis E, Anagnostou E. Retrocollis as the cardinal feature in a de novo ITRP1 variant. Brain Dev. 2022 May;44(5):347–52.
174. Wang L, Hao Y, Yu P, Cao Z, Zhang J, Zhang X, et al. Identification of a Splicing Mutation in ITPR1 via WES in a Chinese Early-Onset Spinocerebellar Ataxia Family. Cerebellum. 2018 Jun;17(3):294–9.
175. Das J, Lilleker J, Shereef H, Ealing J. Missense mutation in the ITPR1 gene presenting with ataxic cerebral palsy: Description of an affected family and literature review. Neurol Neurochir Pol. 2017;51(6):497–500.
176. Casey JP, Hirouchi T, Hisatsune C, Lynch B, Murphy R, Dunne AM, et al. A novel gain-of-function mutation in the ITPR1 suppressor domain causes spinocerebellar ataxia with altered Ca2+ signal patterns. J Neurol. 2017 Jul;264(7):1444–53.
177. van Dijk T, Barth P, Reneman L, Appelhof B, Baas F, Poll-The BT. A de novo missense mutation in the inositol 1,4,5-triphosphate receptor type 1 gene causing severe pontine and cerebellar hypoplasia: Expanding the phenotype of ITPR1-related spinocerebellar ataxia’s. Am J Med Genet A. 2017 Jan;173(1):207–12.
178. Zambonin JL, Bellomo A, Ben-Pazi H, Everman DB, Frazer LM, Geraghty MT, et al. Spinocerebellar ataxia type 29 due to mutations in ITPR1: a case series and review of this emerging congenital ataxia. Orphanet J Rare Dis. 2017 Jun 28;12(1):121.
179. Dahl N, Hu LJ, Chery M, Fardeau M, Gilgenkrantz S, Nivelon-Chevallier A, et al. Myotubular myopathy in a girl with a deletion at Xq27-q28 and unbalanced X inactivation assigns the MTM1 gene to a 600-kb region. Am J Hum Genet. 1995 May;56(5):1108–15.
180. Guiraud-Chaumeil C, Vincent MC, Laporte J, Fardeau M, Samson F, Mandel JL. A mutation in the MTM1 gene invalidates a previous suggestion of nonallelic heterogeneity in X-linked myotubular myopathy. Am J Hum Genet. 1997 Jun;60(6):1542–4.
181. Kimura S, Sugino S, Ohtani Y, Matsukura M, Nishino I, Ikezawa M, et al. Muscle fiber immaturity and inactivity reduce myonecrosis in Duchenne muscular dystrophy. Ann Neurol. 1998 Dec;44(6):967–71.
182. Tanner SM, Orstavik KH, Kristiansen M, Lev D, Lerman-Sagie T, Sadeh M, et al. Skewed X-inactivation in a manifesting carrier of X-linked myotubular myopathy and in her non-manifesting carrier mother. Hum Genet. 1999 Mar;104(3):249–53.
183. Häne BG, Rogers RC, Schwartz CE. Germline mosaicism in X-linked myotubular myopathy. Clin Genet. 1999 Jul;56(1):77–81.
184. Hammans SR, Robinson DO, Moutou C, Kennedy CR, Dennis NR, Hughes PJ, et al. A clinical and genetic study of a manifesting heterozygote with X-linked myotubular myopathy. Neuromuscul Disord. 2000 Feb;10(2):133–7.
185. Tachi N, Kozuka N, Chiba S, Miyaji M, Watanabe I. A double mutation in a patient with X-linked myotubular myopathy. Pediatr Neurol. 2001 Apr;24(4):297–9.
186. Jungbluth H, Sewry CA, Buj-Bello A, Kristiansen M, Ørstavik KH, Kelsey A, et al. Early and severe presentation of X-linked myotubular myopathy in a girl with skewed X-inactivation. Neuromuscul Disord. 2003 Jan;13(1):55–9.
187. Yu S, Manson J, White S, Bourne A, Waddy H, Davis M, et al. X-linked myotubular myopathy in a family with three adult survivors. Clin Genet. 2003 Aug;64(2):148–52.
188. Zanoteli E, Laporte J, Rocha JCC, Kretz C, Oliveira ASB, Mandel JL, et al. Deletion of both MTM1 and MTMR1 genes in a boy with myotubular myopathy. Am J Med Genet A. 2005 Apr 30;134(3):338–40.
189. Cox K, Gattas M, Harvey P, Dolphin C, Friend K, Yu S. X-linked myotubular myopathy: mutation R69C identified in a family with multiple neonatal deaths. Clin Genet. 2005 May;67(5):441–2.
190. Sustersic B, Neubauer D. “Long-term survivor with X-linked myotubular myopathy”. Dev Med Child Neurol. 2005 May;47(5):358–9.
191. Hoffjan S, Thiels C, Vorgerd M, Neuen-Jacob E, Epplen JT, Kress W. Extreme phenotypic variability in a German family with X-linked myotubular myopathy associated with E404K mutation in MTM1. Neuromuscul Disord. 2006 Nov;16(11):749–53.
192. Smets K. X-linked myotubular myopathy and chylothorax. Neuromuscul Disord. 2008 Feb;18(2):183–4.
193. Chang CY, Lin SP, Lin HY, Chuang CK, Ho CS, Su YN. X-linked myotubular myopathy with a novel MTM1 mutation in a Taiwanese child. J Formos Med Assoc. 2008 Dec;107(12):965–70.
194. McCrea HJ, Kretz C, Laporte J, Ment LR. Dementia in a child with myotubular myopathy. Pediatr Neurol. 2009 Jun;40(6):483–5.
195. Bijarnia S, Puri RD, Jain M, Kler N, Roy S, Urtizberea JA, et al. Mutation studies in X-linked myotubular myopathy in three Indian families. Indian J Pediatr. 2010 Apr;77(4):431–3.
196. Chaudhari T, Todd DA, Kent AL, Dopita B, Hallam L, Freckmann ML, et al. Bilateral subdural hygromas and cephalhaematomas in male twins with severe myotubular myopathy caused by a Novel c.431delT (p.Leu144fs) mutation in MTM1 gene. J Paediatr Child Health. 2011 Jan;47(1–2):64–5.
197. Lee IC, Su PH, Chen JY, Hu JM, Lu JJ, Ng YY. Congenital myotubular myopathy with a novel MTM1 gene mutation in a premature infant presenting with ventilator dependency and intrahepatic cholestasis. J Child Neurol. 2012 Jan;27(1):99–104.
198. Vasli N, Laugel V, Böhm J, Lannes B, Biancalana V, Laporte J. Myotubular myopathy caused by multiple abnormal splicing variants in the MTM1 RNA in a patient with a mild phenotype. Eur J Hum Genet. 2012 Jun;20(6):701–4.
199. Gurgel-Giannetti J, Zanoteli E, de Castro Concentino EL, Abath Neto O, Pesquero JB, Reed UC, et al. Necklace fibers as histopathological marker in a patient with severe form of X-linked myotubular myopathy. Neuromuscul Disord. 2012 Jun;22(6):541–5.
200. Fidani L, Karagianni P, Tsakalidis C, Mitsiakos G, Hatziioannidis I, Biancalana V, et al. Identification of a mutation in the MTM1 gene, associated with X-linked myotubular myopathy, in a Greek family. Hippokratia. 2011 Jul;15(3):278–9.
201. Koga H, Miyako K, Suga N, Hidaka T, Takahashi N. Predisposition to subdural hemorrhage in X-linked myotubular myopathy. Pediatr Neurol. 2012 May;46(5):332–4.
202. Motoki T, Fukuda M, Nakano T, Matsukage S, Fukui A, Akiyoshi S, et al. Fatal hepatic hemorrhage by peliosis hepatis in X-linked myotubular myopathy: a case report. Neuromuscul Disord. 2013 Nov;23(11):917–21.
203. Hagiwara S ichiro, Kubota M, Sakaguchi K, Hiwatari E, Kishimoto H, Kagimoto S. Fatal Hepatic Hemorrhage from Peliosis Hepatis with X-linked Myotubular Myopathy. J Pediatr Gastroenterol Nutr. 2015 May;60(5):e45-6.
204. Robb SA, Sewry CA, Dowling JJ, Feng L, Cullup T, Lillis S, et al. Impaired neuromuscular transmission and response to acetylcholinesterase inhibitors in centronuclear myopathies. Neuromuscul Disord. 2011 Jun;21(6):379–86.
205. Tanboon J, Viravan S, Hayashi YK, Nishino I, Sangruchi T. 2 Month-Old Male with Hypotonia. Brain Pathol. 2015 Sep;25(5):651–2.
206. Savarese M, Musumeci O, Giugliano T, Rubegni A, Fiorillo C, Fattori F, et al. Novel findings associated with MTM1 suggest a higher number of female symptomatic carriers. Neuromuscul Disord. 2016;26(4–5):292–9.
207. Kosma K, Mitrakos A, Sofokleous C, Papadimas G, Fryssira H, Kitsiou-Tzeli S, et al. A Female Patient with Xq28 Microduplication Presenting with Myotubular Myopathy, Confirmed with a Custom-Designed X-array. Neuropediatrics. 2019 Feb;50(1):61–3.
208. Nishikawa A, Iida A, Hayashi S, Okubo M, Oya Y, Yamanaka G, et al. Three novel MTM1 pathogenic variants identified in Japanese patients with X-linked myotubular myopathy. Mol Genet Genomic Med. 2019 May;7(5):e621.
209. Funayama K, Shimizu H, Tanaka H, Kawachi I, Nishino I, Matsui K, et al. An autopsy case of peliosis hepatis with X-linked myotubular myopathy. Leg Med (Tokyo). 2019 May;38:77–82.
210. Kraatari M, Tuominen H, Tuupanen S, Haapaniemi T, Moilanen J, Rahikkala E. X-linked myotubular myopathy mimics hereditary spastic paraplegia in two female manifesting carriers of pathogenic MTM1 variant. Eur J Med Genet. 2020 Nov;63(11):104040.
211. Varma U, Mukherjee D, Hughes I, Sethuraman C, Kamupira S. X-Linked Myotubular Myopathy and Duchenne Muscular Dystrophy in a Preterm Infant: A Rare Combination. Pediatrics. 2020 Sep;146(3).
212. Bryen SJ, Oates EC, Evesson FJ, Lu JK, Waddell LB, Joshi H, et al. Pathogenic deep intronic MTM1 variant activates a pseudo-exon encoding a nonsense codon resulting in severe X-linked myotubular myopathy. Eur J Hum Genet. 2021 Jan;29(1):61–6.
213. Gangfuss A, Schmitt D, Roos A, Braun F, Annoussamy M, Servais L, et al. Diagnosing X-linked Myotubular Myopathy - A German 20-year Follow Up Experience. J Neuromuscul Dis. 2021;8(1):79–90.
214. Annoussamy M, Lilien C, Gidaro T, Gargaun E, Chê V, Schara U, et al. X-linked myotubular myopathy: A prospective international natural history study. Neurology. 2019 Apr 16;92(16):e1852–67.
215. Omata K, Okada N, Miyahara G, Hirata Y, Sanada Y, Onishi Y, et al. Peliosis Hepatis in a Child with X-Linked Myotubular Myopathy Treated with Living-Donor Liver Transplant: A Case Report. Transplant Proc. 2021 May;53(4):1317–21.
216. Gómez-González C, Rosas-Alonso R, Rodríguez-Antolín C, García-Guede A, Ibáñez de Caceres I, Sanguino J, et al. Symptomatic heterozygous X-Linked myotubular myopathy female patient with a large deletion at Xq28 and decrease expression of normal allele. Eur J Med Genet. 2021 Apr;64(4):104170.
217. Neese JM, Yum S, Matesanz S, Raffini LJ, Whitworth HB, Loomes KM, et al. Intracranial hemorrhage secondary to vitamin K deficiency in X-linked myotubular myopathy. Neuromuscul Disord. 2021 Jul;31(7):651–5.
218. Molera C, Sarishvili T, Nascimento A, Rtskhiladze I, Muñoz Bartolo G, Fernández Cebrián S, et al. Intrahepatic Cholestasis Is a Clinically Significant Feature Associated with Natural History of X-Linked Myotubular Myopathy (XLMTM): A Case Series and Biopsy Report. J Neuromuscul Dis. 2022;9(1):73–82.
219. Zhao Y, Zhao Z, Shen H, Bing Q, Hu J. Characterization and genetic diagnosis of centronuclear myopathies in seven Chinese patients. Neurol Sci. 2018 Dec;39(12):2043–51.
220. Yabe T, Itonaga T, Kuga S, Koga H, Kusaba T, Nishida H, et al. An autopsy case of recurrent pneumothorax and peliosis-like intrapulmonary hematoma with X-linked myotubular myopathy. Brain Dev. 2022 Mar;44(3):234–8.
221. de Carvalho Nunes G, Grenier K, Maedler Kron C, Kitzler T, Helou J El, Rosenblatt DS, et al. Pulmonary lymphangiectasia in myotubular myopathy: a novel unrecognized association? Neuromuscul Disord. 2022 Jun;32(6):512–5.
222. Bosco L, Leone D, Costa Comellas L, Monforte M, Pane M, Mercuri E, et al. Novel Splicing Mutation in MTM1 Leading to Two Abnormal Transcripts Causes Severe Myotubular Myopathy. Int J Mol Sci. 2022 Sep 7;23(18).
223. El Achkar CM, Rosen Sheidley B, O’Rourke D, Takeoka M, Poduri A. Compound heterozygosity with PRRT2: Pushing the phenotypic envelope in genetic epilepsies. Epilepsy Behav Case Rep. 2017 Jan 1;11:125–8.
224. Becker F, Schubert J, Striano P, Anttonen AK, Liukkonen E, Gaily E, et al. PRRT2-related disorders: Further PKD and ICCA cases and review of the literature. J Neurol. 2013 May 9;260(5):1234–44.
225. Bovenzi R, Schirinzi T, Pierantozzi M, Stefani A, Capuano A, Mercuri NB, et al. Clinical course of paroxysmal dyskinesias throughout pregnancy. Parkinsonism Relat Disord. 2020 Nov 1;80:19–20.
226. Brueckner F, Kohl B, Puest B, Gassner S, Osseforth J, Lindenau M, et al. Unusual variability of PRRT2 linked phenotypes within a family. Eur J Paediatr Neurol. 2014;18(4):540–2.
227. Castelnovo G, Renard D, De Verdal M, Luc J, Thouvenot E, Riant F. Progressive ataxia related to PRRT2 gene mutation. J Neurol Sci. 2016 Aug 15;367:220–1.
228. Castiglioni C, López I, Riant F, Bertini E, Terracciano A. PRRT2 mutation causes paroxysmal kinesigenic dyskinesia and hemiplegic migraine in monozygotic twins. Eur J Paediatr Neurol. 2013 May;17(3):254–8.
229. Dale RC, Gardiner A, Antony J, Houlden H. Familial PRRT2 mutation with heterogeneous paroxysmal disorders including paroxysmal torticollis and hemiplegic migraine. Dev Med Child Neurol. 2012 Oct;54(10):958–60.
230. Dale RC, Gardiner A, Branson JA, Houlden H. Benefit of carbamazepine in a patient with hemiplegic migraine associated with PRRT2 mutation. Dev Med Child Neurol. 2014;56(9):910–910.
231. Dayasiri K, Weerapperuma N, Wright J, Anand G. Paroxysmal kinesigenic dyskinesia: a diagnostic challenge. BMJ Case Rep. 2021 Feb 5;14(2).
232. Dekker MCJ, Chengo R, Kumburu HH, Kamsteeg EJ, Hamel BC. Paroxysmal Kinesigenic Dyskinesia: First Molecularly Confirmed Case from Africa. Tremor and other hyperkinetic movements. 2020;10:1–3.
233. Ebrahimi-Fakhari D, Kang KS, Kotzaeridou U, Kohlhase J, Klein C, Assmann BE, et al. Child Neurology: PRRT2-associated movement disorders and differential diagnoses. Neurology. 2014 Oct 1;83(18):1680–3.
234. Extreia J, Monteiro I, Ferreira A, Rocha S. [Familial paroxysmal kinesigenic dyskinesia. A case description]. An Pediatr (Barc). 2015;82(1):e154–7.
235. Feng HY, Qiao F, Tan J, Zhang X, Hu P, Shi YS, et al. Proline-rich transmembrane protein 2 specifically binds to GluA1 but has no effect on AMPA receptor-mediated synaptic transmission. J Clin Lab Anal. 2022 Feb 1;36(2).
236. Friedman J, Olvera J, Silhavy JL, Gabriel SB, Gleeson JG. Mild paroxysmal kinesigenic dyskinesia caused by PRRT2 missense mutation with reduced penetrance. Neurology. 2012 Aug 28;79(9):946–8.
237. Furukawa G, Negishi Y, Takeuchi T, Ishihara N, Okumura A. Lacosamide for children with paroxysmal kinesigenic dyskinesia. Brain Dev. 2020 Sep 1;42(8):617–20.
238. Fusco C, Russo A, Invernizzi F, Frattini D, Pisani F, Garavaglia B. Novel phenotype in a family with infantile convulsions and paroxysmal choreoathetosis syndrome and PRRT2 gene mutation. Brain Dev. 2014 Feb;36(2):183–4.
239. Ganos C, Mencacci N, Gardiner A, Erro R, Batla A, Houlden H, et al. Paroxysmal Kinesigenic Dyskinesia May Be Misdiagnosed in Co-occurring Gilles de la Tourette Syndrome. Mov Disord Clin Pract. 2014 Apr 1;1(1):84–6.
240. García-Howard M, Herranz-Aguirre M, Moreno-Galarraga L, Urretavizcaya-Martínez M, Alegría-Echauri J, Gorría-Redondo N, et al. Case Report: Benign Infantile Seizures Temporally Associated With COVID-19. Front Pediatr. 2020 Aug 6;8(507):1–5.
241. Geng JH, Zheng Y, Li QF, Hou Q, Wang XH, Jiang Y. Case Report: A Case of Concomitant Paroxysmal Kinesigenic Dyskinesia and Epilepsy: Can We Treat Two Birds With One Stone? Front Neurol. 2022 Feb 2;13(826897):1–5.
242. Holm-Yildiz S, Jeppesen TD. Paroksystisk dyskinesi. Ugeskr Laeger. 2021 Jul 19;183(29).
243. I DV, Aysina VA. [The coincidence of benign non-familial infantile seizures type 2 with osteogenesis imperfecta type 1]. SS Korsakov Journal of Neurology and Psychiatry. 2022;122(5):128–31.
244. Igarashi A, Okumura A, Shimojima K, Abe S, Ikeno M, Shimizu T, et al. Focal seizures and epileptic spasms in a child with Down syndrome from a family with a PRRT2 mutation. Brain Dev. 2016 Jun 1;38(6):597–600.
245. Kim SY, Ahn J, Kwak S, Chang MC. A Child Who Suddenly Freezes While Trying to Cross Crosswalks-Unique Clinical Manifestation of Paroxysmal Kinesigenic Dyskinesia: A Case Report. Children. 2020 Dec 1;7(12):1–4.
246. Kita M, Kuwata Y, Murase N, Akiyama Y, Usui T. A Novel Truncation Mutation of the PRRT2 Gene Resulting in a 16-Amino-Acid Protein Causes Self-inducible Paroxysmal Kinesigenic Dyskinesia. Mov Disord Clin Pract. 2017 Jul 1;4(4):625–8.
247. Komatsu K, Fukumura S, Minagawa K, Nakashima M, Saitsu H. A new case of concurrent existence of PRRT2-associated paroxysmal movement disorders with c.649dup variant and 16p11.2 microdeletion syndrome. Brain Dev. 2022 Aug 1;44(7):474–9.
248. Kunii Y, Matsuda N, Yabe H. A case of paroxysmal kinesigenic dyskinesia which exhibited the phenotype of anxiety disorder. Neuropsychiatr Dis Treat. 2017 Aug 16;13:2181–4.
249. Law CY, Yeung WL, Cheung YF, Chan HF, Fung E, Hui J, et al. A common PRRT2 mutation in familial paroxysmal kinesigenic dyskinesia in Hong Kong: a case series of 16 patients. Hong Kong Med J. 2016 Dec 1;22(6):619–22.
250. Li HF, Chen WJ, Ni W, Wu ZY. Paroxysmal kinesigenic dyskinesia and myotonia congenita in the same family: coexistence of a PRRT2 mutation and two CLCN1 mutations. Neurosci Bull. 2014 Dec 2;30(6):1010–6.
251. Lu JG, Bishop J, Cheyette S, Zhulin IB, Guo S, Sobreira N, et al. A novel PRRT2 pathogenic variant in a family with paroxysmal kinesigenic dyskinesia and benign familial infantile seizures. Cold Spring Harb Mol Case Stud. 2018 Feb 1;4(1).
252. Maini I, Iodice A, Spagnoli C, Salerno GG, Bertani G, Frattini D, et al. Expanding phenotype of PRRT2 gene mutations: A new case with epilepsy and benign myoclonus of early infancy. Eur J Paediatr Neurol. 2016 May 1;20(3):454–6.
253. Marano M, Motolese F, Di Lazzaro V, Consoli F, De Luca A. Paroxysmal Dyskinesias in a PRRT2 Mutation Carrier. Tremor and other hyperkinetic movements . 2018;8:616.
254. Martorell L, Macaya A, Pérez-Dueñas B, Ortigoza-Escobar JD. Acetazolamide Improves Episodic Ataxia in a Patient with Non-Verbal Autism and Paroxysmal Dyskinesia Due To PRRT2 Biallelic Variants. Mov Disord Clin Pract. 2022 Oct 1;9(7):979–82.
255. Mathot M, Lederer D, Gerard S, Gueulette E, Deprez M. [PRRT2 mutation and infantile convulsions]. Arch Pediatr. 2017 Oct 1;24(10):1010–2.
256. Matsumoto N, Takahashi S, Okayama A, Araki A, Azuma H. Benign infantile convulsion as a diagnostic clue of paroxysmal kinesigenic dyskinesia: a case series. J Med Case Rep. 2014 Jun 1;8(1).
257. Méneret A, Grabli D, Depienne C, Gaudebout C, Picard F, Dürr A, et al. PRRT2 mutations. Neurology. 2012 Jul 10;79(2):170–4.
258. Ng JKY, Sadia R, Ram D, Jones EA, Biswas S. Expanding the phenotypic spectrum of pathogenic variants in the PRRT2 gene: bilateral papilledema and abducens nerve palsies secondary to pseudotumor cerebri syndrome. J AAPOS. 2021 Dec 1;25(6):364–6.
259. Prabhakara S, Anbazhagan K. Molecular analysis of PRRT2 gene in a case of paroxysmal kinesigenic dyskinesia patient. Ann Indian Acad Neurol. 2014 Oct 1;17(4):459–62.
260. Suzuki-Muromoto S, Kosaki R, Kosaki K, Kubota M. Familial hemiplegic migraine with a PRRT2 mutation: Phenotypic variations and carbamazepine efficacy. Brain Dev. 2020 Mar 1;42(3):293–7.
261. Tanabe Y, Taira T, Shimotake A, Inoue T, Awaya T, Kato T, et al. [An adult female with proline-rich transmembrane protein 2 related paroxysmal disorders manifesting paroxysmal kinesigenic choreoathetosis and epileptic seizures]. Rinsho Shinkeigaku. 2019;59(3):144–8.
262. Torisu H, Watanabe K, Shimojima K, Sugawara M, Sanefuji M, Ishizaki Y, et al. Girl with a PRRT2 mutation and infantile focal epilepsy with bilateral spikes. Brain Dev. 2014;36(4):342–5.
263. Tran HT, Nguyen K V., Vercueil L. Successful Treatment of a Paroxysmal Kinesigenic Dyskinesia Patient with Carbamazepine-Induced Stevens-Johnson Syndrome Using Oxcarbazepine Monotherapy: A Case Report. Case Rep Neurol. 2021 Sep 13;13(3):598–604.
264. Vergara D, Rubilar C, Witting S, Troncoso M, Caraballo R. Super-refractory status epilepticus related to COVID-19 in a paediatric patient with PRRT2 mutation. Epileptic Disord. 2021 Dec 1;23(6):951–3.
265. Vlachou V, Chu V, Pavlidou E, Ismayilova N, Kshitij M, Kinali M. Benign Infantile Epilepsy Mimicking Reflex Anoxic Seizures in an Infant with PRRT2 Gene Mutation. Indian J Pediatr. 2018 Aug 1;85(8):690.
266. Wang K, Zhao X, Du Y, He F, Peng G, Luo B. Phenotypic overlap among paroxysmal dyskinesia subtypes: Lesson from a family with PRRT2 gene mutation. Brain Dev. 2013 Aug;35(7):664–6.
267. Weber A, Kreth J, Müller U. Intronic PRRT2 mutation generates novel splice acceptor site and causes paroxysmal kinesigenic dyskinesia with infantile convulsions (PKD/IC) in a three generation family. BMC Med Genet. 2016 Mar 3;17:16.
268. Zhang LM, An Y, Pan G, Ding YF, Zhou YF, Yao YH, et al. Reduced Penetrance of PRRT2 Mutation in a Chinese Family With Infantile Convulsion and Choreoathetosis Syndrome. J Child Neurol. 2015 Sep 18;30(10):1263–9.
269. Bayram N, Kaçar Bayram A, Daimagüler HS, Dafsari HS, Bamborschke D, Uyanik G, et al. Genotype-phenotype correlations in ocular manifestations of Marinesco-Sjögren syndrome: Case report and literature review. Eur J Ophthalmol. 2022 May;32(3):NP92–7.
270. Nair P, Hamzeh AR, Mohamed M, Tawfiq N, Al-Ali MT, Bastaki F. Marinesco-Sjögren Syndrome in an Emirati Child with a Novel Mutation in SIL1 Affecting the 5’ Untranslated Region. Med Princ Pract. 2016;25(6):580–2.
271. Gai N, Jiang C, Zou YY, Zheng Y, Liang DS, Wu LQ. Novel SIL1 nonstop mutation in a Chinese consanguineous family with Marinesco-Sjögren syndrome and Dandy-Walker syndrome. Clin Chim Acta. 2016 Jul 1;458:1–4.
272. Cerami C, Tarantino P, Cupidi C, Annesi G, Lo Re V, Gagliardi M, et al. Marinesco-Sjögren syndrome caused by a new SIL1 frameshift mutation. J Neurol Sci. 2015 Jul 15;354(1–2):112–3.
273. Byrne S, Dlamini N, Lumsden D, Pitt M, Zaharieva I, Muntoni F, et al. SIL1-related Marinesco-Sjoegren syndrome (MSS) with associated motor neuronopathy and bradykinetic movement disorder. Neuromuscul Disord. 2015 Jul;25(7):585–8.
274. Terracciano A, Renaldo F, Zanni G, D’Amico A, Pastore A, Barresi S, et al. The use of muscle biopsy in the diagnosis of undefined ataxia with cerebellar atrophy in children. Eur J Paediatr Neurol. 2012 May;16(3):248–56.
275. Fujitake J, Komatsu Y, Hataya Y, Nishikawa A, Eriguchi M, Mizuta H, et al. A case of Marinesco-Sjögren syndrome: MRI observations of skeletal muscles, bone metabolism, and treatment with testosterone and risedronate. Intern Med. 2011;50(2):145–9.
276. Varho TT, Alajoki LE, Posti KM, Korhonen TT, Renlund MG, Nyman SRG, et al. Phenotypic spectrum of Salla disease, a free sialic acid storage disorder. Pediatr Neurol. 2002 Apr;26(4):267–73.
277. Debray FG, Lefebvre C, Colinet S, Segers K, Stevens R. Free sialic acid storage disease mimicking cerebral palsy and revealed by blood smear examination. J Pediatr. 2011 Jan;158(1):165, 165.e1.
278. Martin RA, Slaugh R, Natowicz M, Pearlman K, Orvisky E, Krasnewich D, et al. Sialic acid storage disease of the Salla phenotype in American monozygous twin female sibs. Am J Med Genet A. 2003 Jul 1;120A(1):23–7.
279. Landau D, Cohen D, Shalev H, Pinsk V, Yerushalmi B, Zeigler M, et al. A novel mutation in the SLC17A5 gene causing both severe and mild phenotypes of free sialic acid storage disease in one inbred Bedouin kindred. Mol Genet Metab. 2004 Jun;82(2):167–72.
280. Strauss KA, Puffenberger EG, Craig DW, Panganiban CB, Lee AM, Hu-Lince D, et al. Genome-wide SNP arrays as a diagnostic tool: clinical description, genetic mapping, and molecular characterization of Salla disease in an Old Order Mennonite population. Am J Med Genet A. 2005 Oct 15;138A(3):262–7.
281. Mochel F, Engelke UFH, Barritault J, Yang B, McNeill NH, Thompson JN, et al. Elevated CSF N-acetylaspartylglutamate in patients with free sialic acid storage diseases. Neurology. 2010 Jan 26;74(4):302–5.
282. Linnankivi T, Lönnqvist T, Autti T. A case of Salla disease with involvement of the cerebellar white matter. Neuroradiology. 2003 Feb;45(2):107–9.
283. Biancheri R, Rossi A, Verbeek HA, Schot R, Corsolini F, Assereto S, et al. Homozygosity for the p.K136E mutation in the SLC17A5 gene as cause of an Italian severe Salla disease. Neurogenetics. 2005 Dec;6(4):195–9.
284. Biancheri R, Verbeek E, Rossi A, Gaggero R, Roccatagliata L, Gatti R, et al. An Italian severe Salla disease variant associated with a SLC17A5 mutation earlier described in infantile sialic acid storage disease. Clin Genet. 2002 Jun;61(6):443–7.
285. Kleta R, Morse RP, Orvisky E, Krasnewich D, Alroy J, Ucci AA, et al. Clinical, biochemical, and molecular diagnosis of a free sialic acid storage disease patient of moderate severity. Mol Genet Metab. 2004 Jun;82(2):137–43.
286. Kleta R, Aughton DJ, Rivkin MJ, Huizing M, Strovel E, Anikster Y, et al. Biochemical and molecular analyses of infantile free sialic acid storage disease in North American children. Am J Med Genet A. 2003 Jul 1;120A(1):28–33.
287. Parazzini C, Arena S, Marchetti L, Menni F, Filocamo M, Verheijen FW, et al. Infantile sialic acid storage disease: serial ultrasound and magnetic resonance imaging features. AJNR Am J Neuroradiol. 2003 Mar;24(3):398–400.
288. Sønderby Christensen P, Kaad PH, Ostergaard JR. Two cases of Salla disease in Danish children. Acta Paediatr. 2003 Nov;92(11):1357–8.
289. Mochel F, Yang B, Barritault J, Thompson JN, Engelke UFH, McNeill NH, et al. Free sialic acid storage disease without sialuria. Ann Neurol. 2009 Jun;65(6):753–7.
290. Couce ML, Macías-Vidal J, Castiñeiras DE, Bóveda MD, Fraga JM, Fernández-Marmiesse A, et al. The early detection of Salla disease through second-tier tests in newborn screening: how to face incidental findings. Eur J Med Genet. 2014 Sep;57(9):527–31.
291. Barmherzig R, Bullivant G, Cordeiro D, Sinasac DS, Blaser S, Mercimek-Mahmutoglu S. A New Patient With Intermediate Severe Salla Disease With Hypomyelination: A Literature Review for Salla Disease. Pediatr Neurol. 2017 Sep;74:87-91.e2.
292. Kang E, Kim YM, Heo SH, Jung E, Kim KS, Yoo HJ, et al. Biochemical and molecular analyses of infantile sialic acid storage disease in a patient with nonimmune hydrops fetalis. Clin Chim Acta. 2018 Jul;482:199–202.
293. Akasaka M, Kamei A, Araya N, Oyama K, Sasaki M. Characteristic proton magnetic resonance spectroscopy in glucose transporter type 1 deficiency syndrome. Pediatr Int. 2018 Oct 1;60(10):978–9.
294. Algahtani H, Shirah B, Albarakaty A, Al-Qahtani MH, Abdulkareem AA, Naseer MI. A Novel Intronic Variant in SLC2A1 Gene in a Saudi Patient with Myoclonic Epilepsy. J Epilepsy Res. 2020 Jun 30;10(1):40–3.
295. Almuqbil M, Rivkin MJ, Takeoka M, Yang E, Rodan LH. Transient regional cerebral hypoperfusion during a paroxysmal hemiplegic event in GLUT1 deficiency syndrome. Eur J Paediatr Neurol. 2018 May 1;22(3):544–7.
296. Anand G, Padeniya A, Hanrahan D, Scheffer H, Zaiwalla Z, Cox D, et al. Milder phenotypes of glucose transporter type 1 deficiency syndrome. Dev Med Child Neurol. 2011 Jul;53(7):664–8.
297. Angeli M, Vergadi E, Niotakis G, Raissaki M, Galanakis E. Abnormal gait and hypoglycorrhachia in a toddler with seizures. Pediatr Investig. 2022 Mar 1;6(1):47–9.
298. Anheim M, Maillart E, Vuillaumier-Barrot S, Flamand-Rouvière C, Pineau F, Ewenczyk C, et al. Excellent response to acetazolamide in a case of paroxysmal dyskinesias due to GLUT1-deficiency. J Neurol. 2011 Feb;258(2):316–7.
299. Appavu B, Mangum T, Obeid M. Glucose Transporter 1 Deficiency: A Treatable Cause of Opsoclonus and Epileptic Myoclonus. Pediatr Neurol. 2015 Oct 1;53(4):364–6.
300. Baschieri F, Batla A, Erro R, Ganos C, Cordivari C, Bhatia KP. Paroxysmal exercise-induced dystonia due to GLUT1 mutation can be responsive to levodopa: a case report. J Neurol. 2014;261(3):615–6.
301. Bawazir WM, Gevers EF, Flatt JF, Ang AL, Jacobs B, Oren C, et al. An infant with pseudohyperkalemia, hemolysis, and seizures: cation-leaky GLUT1-deficiency syndrome due to a SLC2A1 mutation. J Clin Endocrinol Metab. 2012 Jun;97(6):e987–93.
302. Bovi T, Fasano A, Juergenson I, Gellera C, Castellotti B, Fontana E, et al. Paroxysmal exercise-induced dyskinesia with self-limiting partial epilepsy: a novel GLUT-1 mutation with benign phenotype. Parkinsonism Relat Disord. 2011 Jul;17(6):479–81.
303. Brockmann K, Wang D, Korenke CG, Von Moers A, Ho YY, Pascual JM, et al. Autosomal dominant glut-1 deficiency syndrome and familial epilepsy. Ann Neurol. 2001;50(4):476–85.
304. Byrne S, Kearns J, Carolan R, Mc Menamin J, Klepper J, Webb D. Refractory absence epilepsy associated with GLUT-1 deficiency syndrome. Epilepsia. 2011 May;52(5):1021–4.
305. Bzduch V, Sykora P, Behulova D, Kolnikova M, Klcova V. Glucose transporter type 1 (GLUT-1) deficiency. Bratislava Medical Journal. 2008;109(5):245.
306. Chambon R, Vuillaumier-Barrot S, Seta N, Wagner S, Sarret C. Partial effectiveness of acetazolamide in a mild form of GLUT1 deficiency: a pediatric observation. Mov Disord. 2013 Oct;28(12):1749–51.
307. Çolak R, Alkan Özdemir S, Yangın Ergon E, Kağnıcı M, Çalkavur Ş. A Different SLC2A1 Gene Mutation in Glut 1 Deficiency Syndrome: c.734A>C. Balkan Med J. 2017 Nov 1;34(6):580–3.
308. Coman DJ, Sinclair KG, Burke CJ, Appleton DB, Pelekanos JT, O’Neil CM, et al. Seizures, ataxia, developmental delay and the general paediatrician: glucose transporter 1 deficiency syndrome. J Paediatr Child Health. 2006 May;42(5):263–7.
309. Cornejo E V, Cabello A JF, Colombo C M, Raimann B E. [Glucose transponer type 1 deficiency síndrome (GLUT-1 SD) treated with ketogenic diet. Report of one case]. Rev Med Chil. 2007 May;135(5):631–5.
310. Daoudi S, Lounis M, Chibout S, Bensaadi N, Ait-kaci-Ahmed M. [De Vivo disease. GLUT-1 deficiency syndrome: a case report]. Arch Pediatr. 2014 Mar;21(3):302–5.
311. Diaz J, Fonseca AG, Arboleda R, Frade A, Gennaro MP, Jayakar P, et al. Case Report: The Association of Wilson Disease in a Patient With Ataxia and GLUT-1 Deficiency. Front Pediatr. 2021 Oct 5;9:750593.
312. Diaz-Arias LA, Henry-Barron BJ, Buchholz A, Cervenka MC. Positive impact of a modified Atkins diet on cognition, seizure control, and abnormal movements in an adult with glucose transporter type 1 deficiency syndrome: case report. Neurol Sci. 2022 May 1;43(5):3449–52.
313. Dozières-Puyravel B, Zaman S, Petrou S, François L, Vuillaumier-Barrot S, Mochel F, et al. Usefulness of diagnostic tools in a GLUT1 deficiency syndrome patient with 2 inherited mutations. Brain Dev. 2019 Oct 1;41(9):808–11.
314. Friedman JRL, Thiele EA, Wang D, Levine KB, Cloherty EK, Natowicz MR. Atypical GLUT1 deficiency with prominent movement disorder responsive to ketogenic diet. Mov Disord. 2006;21(2):241–4.
315. Fung EL wah, Ho YY, Hui J, Wong JH, Ng TB, Fong NYF, et al. First report of GLUT1 deficiency syndrome in Chinese patients with novel and hot spot mutations in SLC2A1 gene. Brain Dev. 2011 Feb;33(2):170–3.
316. Gagliardi S, Davin A, Ricca I, Grieco GS, Zangaglia R, Pierelli F, et al. A new GLUT-1 mutation in a family with glucose transporter 1 deficiency syndrome. Mov Disord. 2012 May;27(6):804–5.
317. Gaspard N, Suls A, Vilain C, De Jonghe P, Van Bogaert P. “Benign” myoclonic epilepsy of infancy as the initial presentation of glucose transporter-1 deficiency. Epileptic Disord. 2011 Sep;13(3):300–3.
318. De Giorgis V, Ferraris C, Brena ML, Farris G, Gentilino V, Guglielmetti M, et al. Classic ketogenic diet in parenteral nutrition in a GLUT1DS patient: Doing more with less in an acute surgical setting. Front Nutr. 2023;10:1114386.
319. Gökben S, Yilmaz S, Klepper J, Serdaroǧlu G, Tekgül H. Video/EEG recording of myoclonic absences in GLUT1 deficiency syndrome with a hot-spot R126C mutation in the SLC2A1 gene. Epilepsy Behav. 2011 Jun;21(2):200–2.
320. Good JM, Atallah I, Jimenez MC, Benninger D, Kuntzer T, Superti-Furga A, et al. NGS-Based Diagnosis of Treatable Neurogenetic Disorders in Adults: Opportunities and Challenges. Genes (Basel). 2021;12(5).
321. Gowda VK, Sheshu S. Intermittent Ataxia with Early Onset Absence Epilepsy in Glucose Transporter Type 1 Deficiency Syndrome. Indian Pediatr. 2015 Nov;52(11):997.
322. Graham JM. GLUT1 deficiency syndrome as a cause of encephalopathy that includes cognitive disability, treatment-resistant infantile epilepsy and a complex movement disorder. Eur J Med Genet. 2012 May;55(5):332–4.
323. Gramer G, Wolf NI, Vater D, Bast T, Santer R, Kamsteeg EJ, et al. Glucose transporter-1 (GLUT1) deficiency syndrome: diagnosis and treatment in late childhood. Neuropediatrics. 2012;43(3):168–71.
324. Haberlandt E, Karall D, Jud V, Baumgartner SS, Zotter S, Rostasy K, et al. Glucose transporter type 1 deficiency syndrome effectively treated with modified Atkins diet. Neuropediatrics. 2014;45(2):117–9.
325. Harris MLO, Patel H, Garg BP. Intractable seizures, developmental delay, and the ketogenic diet. Semin Pediatr Neurol. 2008 Dec;15(4):209–11.
326. Ho YY, Yang H, Klepper J, Fischbarg J, Wang D, De Vivo DC. Glucose transporter type 1 deficiency syndrome (Glut1DS): methylxanthines potentiate GLUT1 haploinsufficiency in vitro. Pediatr Res. 2001;50(2):254–60.
327. Hoshino H, Takayama K, Ishii A, Takahashi Y, Kanemura H. Glucose transporter type 1 deficiency syndrome associated with autoantibodies to glutamate receptors. Brain Dev. 2020 Oct 1;42(9):686–90.
328. Hu Q, Shen Y, Su T, Liu Y, Xu S. Clinical and Genetic Characteristics of Chinese Children With GLUT1 Deficiency Syndrome: Case Report and Literature Review. Front Genet. 2021 Nov 22;12:734481.
329. Ismayilova N, Hacohen Y, MacKinnon AD, Elmslie F, Clarke A. GLUT-1 deficiency presenting with seizures and reversible leukoencephalopathy on MRI imaging. Eur J Paediatr Neurol. 2018 Nov 1;22(6):1161–4.
330. Ito S, Oguni H, Ito Y, Ishigaki K, Ohinata J, Osawa M. Modified Atkins diet therapy for a case with glucose transporter type 1 deficiency syndrome. Brain Dev. 2008 Mar;30(3):226–8.
331. Ito Y, Gertsen E, Oguni H, Nakayama T, Matsuo M, Funatsuka M, et al. Clinical presentation, EEG studies, and novel mutations in two cases of GLUT1 deficiency syndrome in Japan. Brain Dev. 2005;27(4):311–7.
332. Joshi C, Greenberg CR, De Vivo D, Wang D, Chan-Lui W, Booth FA. GLUT1 deficiency without epilepsy: yet another case. J Child Neurol. 2008 Jul;23(7):832–4.
333. Juozapaite S, Praninskiene R, Burnyte B, Ambrozaityte L, Skerliene B. Novel mutation in a patient with late onset GLUT1 deficiency syndrome. Brain Dev. 2017 Apr 1;39(4):352–5.
334. Kitamura Y, Okumura A, Hayashi M, Mori H, Takahashi S, Yanagihara K, et al. Oxidative stress markers and phosphorus magnetic resonance spectroscopy in a patient with GLUT1 deficiency treated with modified Atkins diet. Brain Dev. 2012 May;34(5):372–5.
335. Klepper J, Scheffer H, Elsaid MF, Kamsteeg EJ, Leferink M, Ben-Omran T. Autosomal recessive inheritance of GLUT1 deficiency syndrome. Neuropediatrics. 2009;40(5):207–10.
336. Klepper J, Leiendecker B, Heussinger N, Lausch E, Bosch F. Severe Hypertriglyceridemia in Glut1D on Ketogenic Diet. Neuropediatrics. 2016 Apr 1;47(2):132–5.
337. Klepper J, Engelbrecht V, Scheffer H, van der Knaap MS, Fiedler A. GLUT1 deficiency with delayed myelination responding to ketogenic diet. Pediatr Neurol. 2007 Aug;37(2):130–3.
338. Klepper J, Willemsen M, Verrips A, Guertsen E, Herrmann R, Kutzick C, et al. Autosomal dominant transmission of GLUT1 deficiency. Hum Mol Genet. 2001 Jan 1;10(1):63–8.
339. Kolic I, Nisevic JR, Cicvaric IV, Ahel IB, Tomulic KL, Segulja S, et al. GLUT1 Deficiency Syndrome-Early Treatment Maintains Cognitive Development? (Literature Review and Case Report). Genes (Basel). 2021 Sep 1;12(9).
340. Koy A, Assmann B, Klepper J, Mayatepek E. Glucose transporter type 1 deficiency syndrome with carbohydrate-responsive symptoms but without epilepsy. Dev Med Child Neurol. 2011 Dec;53(12):1154–6.
341. Kraoua I, Benrhouma H, Vuillaumier-Barrot S, Klaa H, Youssef-Turki I Ben. A Case of Progressive Chorea Resulting From GLUT1 Deficiency. Mov Disord Clin Pract. 2015 Dec 1;2(4):424–5.
342. Lee MS, Kim YJ, Kim EJ, Lee MJ. Overlap of autism spectrum disorder and glucose transporter 1 deficiency syndrome associated with a heterozygous deletion at the 1p34.2 region. J Neurol Sci. 2015 Sep 15;356(1–2):212–4.
343. Levy B, Wang D, Ullner PM, Engelstad K, Yang H, Nahum O, et al. Uncovering microdeletions in patients with severe Glut-1 deficiency syndrome using SNP oligonucleotide microarray analysis. Mol Genet Metab. 2010 Jun;100(2):129–35.
344. Li Z, Han C, Chen G, Zhao H. [Analysis of clinical phenotype and variant of SLC2A1 gene in a Chinese pedigree affected with glucose transporter 1 deficiency syndrome]. Zhonghua Yi Xue Yi Chuan Xue Za Zhi. 2022 Aug 10;39(8):884–8.
345. Liu Y, Bao X, Wang D, Fu N, Zhang X, Cao G, et al. Allelic variations of glut-1 deficiency syndrome: the chinese experience. Pediatr Neurol. 2012 Jul;47(1):30–4.
346. Logel SN, Connor EL, Hsu DA, Fenske RJ, Paloian NJ, De Vivo DC. Exploring diazoxide and continuous glucose monitoring as treatment for Glut1 deficiency syndrome. Ann Clin Transl Neurol. 2021 Nov 1;8(11):2205–9.
347. Madaan P, Jauhari P, Chakrabarty B, Gulati S. Jeavons syndrome in a family with GLUT1-deficiency syndrome. Seizure. 2019 Oct 1;71:158–60.
348. Magrinelli F, Mulroy E, Schneider SA, Latorre A, Di Lazzaro G, Hennig A, et al. Criss-cross gait: A clue to glucose transporter type 1 deficiency syndrome. Neurology. 2020 Sep 15;95(11):500–1.
349. Messana T, Russo A, Vergaro R, Boni A, Santucci M, Pini A. Glucose Transporter Type 1 Deficiency Syndrome: Developmental Delay and Early-Onset Ataxia in a Novel Mutation of the SLC2A1 Gene. J Pediatr Neurosci. 2018 Oct 1;13(4):496–9.
350. Mohammad SS, Coman D, Calvert S. Glucose transporter 1 deficiency syndrome and hemiplegic migraines as a dominant presenting clinical feature. J Paediatr Child Health. 2014 Dec 1;50(12):1025–6.
351. Nakagama Y, Isojima T, Mizuno Y, Takahashi N, Kitanaka S, Igarashi T. Growth hormone deficiency: a possible complication of glucose transporter 1 deficiency? Acta Paediatr. 2012 Jun;101(6).
352. Nakamura S, Osaka H, Muramatsu S, Aoki S, Jimbo EF, Yamagata T. Mutational and functional analysis of Glucose transporter I deficiency syndrome. Mol Genet Metab. 2015 Nov 1;116(3):157–62.
353. Narváez C, Lacaux P, Cortés C, Manterola C, Carrasco X. Phenotypic variability of GLUT1 deficiency: When is necessary to suspect? Rev Chil Pediatr. 2020 Mar 1;91(2):260–4.
354. Neville BGR, Besag MC, Marsden CD, Besag FMC. Exercise induced steroid dependent dystonia, ataxia, and alternating hemiplegia associated with epilepsy. J Neurol Neurosurg Psychiatry. 1998;65:241–4.
355. Nicita F, Schirinzi T, Stregapede F, Vasco G, Bertini E, Travaglini L. SLC2A1 mutations are a rare cause of pediatric-onset hereditary spastic paraplegia. Eur J Paediatr Neurol. 2019 Mar 1;23(2):329–32.
356. Overweg-Plandsoen WCG, Groener JEM, Wang D, Onkenhout W, Brouwer OF, Bakker HD, et al. GLUT-1 deficiency without epilepsy--an exceptional case. J Inherit Metab Dis. 2003;26(6):559–63.
357. Panandikar GA, Ravat SH, Ansari RR, Desai KM. Rare and Treatable Cause of Early-Onset Refractory Absence Seizures. J Pediatr Neurosci. 2018 Jul 1;13(3):358–61.
358. Parolin G, Drigo P, Toldo I, Boniver C, Gatta M, Burlina A, et al. Pre- and postprandial electroencephalography in glucose transporter type 1 deficiency syndrome: an illustrative case to discuss the concept of carbohydrate responsiveness. J Child Neurol. 2011 Jan;26(1):103–8.
359. Pawlik W, Okulewicz P, Pawlik J, Krzywińska-Zdeb E. Diagnostic and Clinical Manifestation Differences of Glucose Transporter Type 1 Deficiency Syndrome in a Family with SLC2A1 Gene Mutation. Int J Environ Res Public Health. 2022 Mar 1;19(6).
360. Pearson TS, Pons R, Engelstad K, Kane SA, Goldberg ME, De Vivo DC. Paroxysmal eye-head movements in Glut1 deficiency syndrome. Neurology. 2017 Apr 25;88(17):1666–73.
361. Pellegrin S, Cantalupo G, Opri R, Dalla Bernardina B, Darra F. EEG findings during “paroxysmal hemiplegia” in a patient with GLUT1-deficiency. Eur J Paediatr Neurol. 2017 May 1;21(3):580–2.
362. Pérez-Dueñas B, Prior C, Ma Q, Fernández-Álvarez E, Setoain X, Artuch R, et al. Childhood chorea with cerebral hypotrophy: a treatable GLUT1 energy failure syndrome. Arch Neurol. 2009 Nov;66(11):1410–4.
363. Posar A, Santucci M. Unusual phenotype of glucose transport protein type 1 deficiency syndrome: A case report and literature review. J Pediatr Neurosci. 2014;9(1):36–8.
364. Ragona F, Matricardi S, Castellotti B, Patrini M, Freri E, Binelli S, et al. Refractory absence epilepsy and glut1 deficiency syndrome: a new case report and literature review. Neuropediatrics. 2014 Oct 1;45(5):328–32.
365. Reis S, Matias J, Machado R, Monteiro JP. Paroxysmal ocular movements - an early sign in Glut1 deficiency Syndrome. Metab Brain Dis. 2018 Aug 1;33(4):1381–3.
366. Roulet-Perez E, Ballhausen D, Bonafé L, Cronel-Ohayon S, Maeder-Ingvar M. Glut-1 deficiency syndrome masquerading as idiopathic generalized epilepsy. Epilepsia. 2008 Nov;49(11):1955–8.
367. van Samkar A, Leen WG, Willemsen MAAP, Verrips A. Hypointensity of the Basal Ganglia in Adults with Glucose Transporter Protein Type 1 Deficiency Syndrome: A Novel Magnetic Resonance Imaging Finding. Ann Neurol. 2020 Jan 1;87(1):10–1.
368. Schneider SA, Paisan-Ruiz C, Garcia-Gorostiaga I, Quinn NP, Weber YG, Lerche H, et al. GLUT1 gene mutations cause sporadic paroxysmal exercise-induced dyskinesias. Mov Disord. 2009 Aug 15;24(11):1684–8.
369. Scoppola C, Magli G, Conti M, Fadda M, Luzzu GM, Simula DM, et al. CACNA1A-Linked Hemiplegic Migraine in GLUT 1 Deficiency Syndrome: A Case Report. Front Neurol. 2021 May 31;12.
370. Sen S, Keough K, Gibson J. Clinical reasoning: novel GLUT1-DS mutation: refractory seizures and ataxia. Neurology. 2015 Apr 14;84(15):e111–4.
371. Shibata T, Kobayashi K, Yoshinaga H, Ono H, Shinpo M, Kagitani-Shimono K. Another Case of Glucose Transporter 1 Deficiency Syndrome with Periventricular Calcification, Cataracts, Hemolysis, and Pseudohyperkalemia. Neuropediatrics. 2017 Oct 1;48(5):390–3.
372. Shiohama T, Fujii K, Takahashi S, Nakamura F, Kohno Y. Reversible white matter lesions during ketogenic diet therapy in glucose transporter 1 deficiency syndrome. Pediatr Neurol. 2013 Dec;49(6):493–6.
373. Slaughter L, Vartzelis G, Arthur T. New GLUT-1 mutation in a child with treatment-resistant epilepsy. Epilepsy Res. 2009 Apr;84(2–3):254–6.
374. Soliani L, Martorell L, Yubero D, Verges C, Petit V, Ortigoza-Escobar JD. Paroxysmal Non-Kinesigenic Dyskinesia: Utility of the Quantification of GLUT1 in Red Blood Cells. Mov Disord Clin Pract. 2021 Feb 1;9(2):252–4.
375. Takahashi S, Matsufuji M, Yonee C, Tsuru H, Sano N, Oguni H. Somatic mosaicism for a SLC2A1 mutation: implications for genetic counseling for GLUT1 deficiency syndrome. Clin Genet. 2017 Jun 1;91(6):932–3.
376. Takahashi S, Ohinata J, Suzuki N, Amamiya S, Kajihama A, Sugai R, et al. Molecular analysis and anticonvulsant therapy in two patients with glucose transporter 1 deficiency syndrome: a successful use of zonisamide for controlling the seizures. Epilepsy Res. 2008 Jul;80(1):18–22.
377. Tchapyjnikov D, Mikati MA. Acetazolamide-responsive Episodic Ataxia Without Baseline Deficits or Seizures Secondary to GLUT1 Deficiency: A Case Report and Review of the Literature. Neurologist. 2018;23(1):17–8.
378. Thouin A, Crompton DE. Glut1 deficiency syndrome: Absence epilepsy and La Soupe du Jour. Pract Neurol. 2016 Feb 1;16(1):50–2.
379. Tornese G, Patti G, Pellegrin MC, Costa P, Faletra F, Faleschini E, et al. A case report of glucose transporter 1 deficiency syndrome with growth hormone deficiency diagnosed before starting ketogenic diet. Ital J Pediatr. 2020 Aug 26;46(1).
380. Urbizu A, Cuenca-León E, Raspall-Chaure M, Gratacòs M, Conill J, Redecillas S, et al. Paroxysmal exercise-induced dyskinesia, writer’s cramp, migraine with aura and absence epilepsy in twin brothers with a novel SLC2A1 missense mutation. J Neurol Sci. 2010 Aug 15;295(1–2):110–3.
381. Üstyol A, Takahashi S, Hatipoğlu HU, Duman MA, Elevli M, Duru HNS. A novel mutation in SLC2A1 gene causing GLUT-1 deficiency syndrome in a young adult patient. Turk J Pediatr. 2019;61(6):946–8.
382. Vermeer S, Koolen DA, Visser G, Brackel HJL, van der Burgt I, de Leeuw N, et al. A novel microdeletion in 1(p34.2p34.3), involving the SLC2A1 (GLUT1) gene, and severe delayed development. Dev Med Child Neurol. 2007 May;49(5):380–4.
383. Vieker S, Schmitt J, Längler A, Schmidt W, Klepper J. Unusual sensitivity to steroid treatment in intractable childhood epilepsy suggests GLUT1 deficiency syndrome. Neuropediatrics. 2012;43(5):275–8.
384. De Vivo DC, Trifiletti RR, Jacobson RI, Ronen GM, Behmand RA, Harik SI. Defective Glucose Transport across the Blood-Brain Barrier as a Cause of Persistent Hypoglycorrhachia, Seizures, and Developmental Delay. N Engl J Med. 2010 Jan 14;325(10):703–9.
385. Wang D, Yang H, Shi L, Ma L, Fujii T, Engelstad K, et al. Functional studies of the T295M mutation causing Glut1 deficiency: glucose efflux preferentially affected by T295M. Pediatr Res. 2008 Nov;64(5):538–43.
386. Weber YG, Storch A, Wuttke T V., Brockmann K, Kempfle J, Maljevic S, et al. GLUT1 mutations are a cause of paroxysmal exertion-induced dyskinesias and induce hemolytic anemia by a cation leak. J Clin Invest. 2008 Jun 2;118(6):2157–68.
387. Wei Z, Wang L, Deng Y. Treatment of myoclonic-atonic epilepsy caused by SLC2A1 de novo mutation with ketogenic diet: A case report. Medicine. 2019 May 1;98(18).
388. Weller CM, Leen WG, Neville BGR, Duncan JS, De Vries B, Geilenkirchen MA, et al. A novel SLC2A1 mutation linking hemiplegic migraine with alternating hemiplegia of childhood. Cephalalgia. 2015 Jan 1;35(1):10–5.
389. Wolking S, Becker F, Bast T, Wiemer-Kruel A, Mayer T, Lerche H, et al. Focal epilepsy in Glucose transporter type 1 (Glut1) defects: Case reports and a review of literature. J Neurol. 2014 Jan 1;261(10):1881–6.
390. Woo SB, Lee KH, Kang HC, Yang H, De Vivo DC, Kim SK. First report of glucose transporter 1 deficiency syndrome in Korea with a novel splice site mutation. Gene. 2012 Sep 15;506(2):380–2.
391. Yu M, Miao J, Lv Y, Wang X, Zhang W, Shao N, et al. A Challenging Diagnosis of Atypical Glut1-DS: A Case Report and Literature Review. Front Neurol. 2021 Jan 28;11.
392. Yubero D, O’Callaghan M, Montero R, Ormazabal A, Armstrong J, Espinos C, et al. Association between coenzyme Q10 and glucose transporter (GLUT1) deficiency. BMC Pediatr. 2014 Nov 8;14(1).
393. Namekawa M, Takiyama Y, Sakoe K, Nagaki H, Shimazaki H, Yoshimura M, et al. A Japanese SPG4 family with a novel missense mutation of the SPG4 gene: intrafamilial variability in age at onset and clinical severity. Acta Neurol Scand. 2002 Dec;106(6):387–91.
394. Nicholas AP, O’Hearn E, Holmes SE, Chen DT, Margolis RL. Clinical signs and symptoms in a large hereditary spastic paraparesis pedigree with a novel spastin mutation. Mov Disord. 2004 Jun;19(6):641–8.
395. Yazıcı I, Yıldırım N, Zorlu Y. The coexistence of multiple sclerosis and hereditary spastic paraparesis in a patient. Neurol Int. 2013 Jun 25;5(2):17–9.
396. Di Fabio R, Tessa A, Marcotulli C, Leonardi L, Pierelli F, Santorelli FM, et al. “When atlastin meets spastin”. Clin Genet. 2014 Nov;86(5):504–5.
397. Aulitzky A, Friedrich K, Gläser D, Gastl R, Kubisch C, Ludolph AC, et al. A complex form of hereditary spastic paraplegia in three siblings due to somatic mosaicism for a novel SPAST mutation in the mother. J Neurol Sci. 2014 Dec 15;347(1–2):352–5.
398. Zádori D, Máté A, Róna-Vörös K, Gergev G, Zimmermann A, Nagy N, et al. The clinical manifestations of two novel SPAST mutations. Clin Neurol Neurosurg. 2015 Sep;136:82–5.
399. Wang K, Zhao G. Exon 8-17 deletions of SPAST in a Chinese family with hereditary spastic paraplegia: a case report and literature review. J Neurol Sci. 2015 Oct 15;357(1–2):282–4.
400. Chelban V, Lynch DS, Houlden H, Wood N. Triple trouble: a striking new phenotype or competing genes in a family with hereditary spastic paraplegia. J Neurol. 2016 Jun;263(6):1232–3.
401. Tisher A, Salardini A. A case report of a woman with young onset cognitive impairment associated with hereditary spastic paraplegia due to a mutation in the SPAST gene. J Neurol Sci. 2016 Aug 15;367:131–2.
402. Matthews AM, Tarailo-Graovac M, Price EM, Blydt-Hansen I, Ghani A, Drögemöller BI, et al. A de novo mosaic mutation in SPAST with two novel alternative alleles and chromosomal copy number variant in a boy with spastic paraplegia and autism spectrum disorder. Eur J Med Genet. 2017 Oct;60(10):548–52.
403. Kawarai T, Montecchiani C, Miyamoto R, Gaudiello F, Caltagirone C, Izumi Y, et al. Spastic paraplegia type 4: A novel SPAST splice site donor mutation and expansion of the phenotype variability. J Neurol Sci. 2017 Sep 15;380:92–7.
404. Ogasawara M, Saito T, Koshimizu E, Akasaka N, Sasaki M. A p.Arg499His Mutation in SPAST Is Associated with Infantile Onset Ascending Spastic Paralysis Complicated with Dysarthria and Anarthria. Neuropediatrics. 2019 Dec;50(6):391–4.
405. Sakaguchi Y, Uehara T, Sasaki M, Fujimura K, Kishi K, Kosaki K, et al. Hereditary spastic paraplegia masqueraded by congenital melanocytic nevus syndrome: Dual pathogenesis of germline non-mosaicism and somatic mosaicism. Eur J Med Genet. 2020 Apr;63(4):103803.
406. Cruz-Camino H, Vázquez-Cantú M, Vázquez-Cantú DL, Santos-Guzmán J, Bandala-Jacques A, Gómez-Gutiérrez R, et al. Clinical Characterization of 2 Siblings with a Homozygous SPAST Variant. Am J Case Rep. 2020 May 11;21:e919463.
407. Nan H, Okamoto K, Gao L, Morishima Y, Ichinose Y, Koh K, et al. A Japanese SPG4 Patient with a Confirmed De Novo Mutation of the SPAST Gene. Intern Med. 2020 Sep 15;59(18):2311–5.
408. Angelini C, Goizet C, Said SA, Camu W, Depienne C, Heron B, et al. Evidence of mosaicism in SPAST variant carriers in four French families. Eur J Hum Genet. 2021 Jul;29(7):1158–63.
409. Nan H, Shiraku H, Mizuno T, Takiyama Y. A p.Arg499His mutation in SPAST is associated with infantile-onset complicated spastic paraplegia: a case report and review of the literature. BMC Neurol. 2021 Nov 9;21(1):439.
410. Xu L, Peng Z, Zhou C, Wang J, Luo H, Lu Q, et al. A Chinese Patient with Spastic Paraplegia Type 4 with a De Novo Mutation in the SPAST Gene. Case Rep Genet. 2021;2021:6636855.
411. Akaba Y, Takeguchi R, Tanaka R, Takahashi S. A Complex Phenotype of a Patient with Spastic Paraplegia Type 4 Caused by a Novel Pathogenic Variant in the SPAST Gene. Case Rep Neurol. 2021;13(3):763–71.
412. Neagu AC, Budișteanu M, Gheorghe DC, Mocanu AI, Mocanu H. Rare Gene Mutations in Romanian Hypoacusis Patients: Case Series and a Review of the Literature. Medicina (Kaunas). 2022 Sep 9;58(9).
413. Jamali F, Ghaedi H, Tafakhori A, Alehabib E, Chapi M, Daftarian N, et al. Homozygous Mutation in TWNK Cases Ataxia, Sensorineural Hearing Loss and Optic Nerve Atrophy. Arch Iran Med. 2019 Dec 1;22(12):728–30.
414. Li X, Li L, Sun Y, Lv F, Zhang G, Liu W, et al. Whole exome sequencing reveals two novel compound heterozygous mutations in TWNK as a cause of the hepatocerebral form of mitochondrial DNA depletion syndrome: a case report. BMC Med Genet. 2019 Aug 27;20(1):146.
415. Sukhudyan B, Gevorgyan A, Sarkissian A, Boltshauser E. Expanding phenotype of mitochondrial depletion syndrome in association with TWNK mutations. Eur J Paediatr Neurol. 2019 May;23(3):537–40.
416. Remtulla S, Emilie Nguyen CT, Prasad C, Campbell C. Twinkle-Associated Mitochondrial DNA Depletion. Pediatr Neurol. 2019 Jan;90:61–5.
417. Park MH, Woo HM, Hong Y Bin, Park JH, Yoon BR, Park JM, et al. Recessive C10orf2 mutations in a family with infantile-onset spinocerebellar ataxia, sensorimotor polyneuropathy, and myopathy. Neurogenetics. 2014 Aug;15(3):171–82.
418. Puckett RL, Lorey F, Rinaldo P, Lipson MH, Matern D, Sowa ME, et al. Maple syrup urine disease: further evidence that newborn screening may fail to identify variant forms. Mol Genet Metab. 2010 Jun;100(2):136–42.
419. Wang YP, Qi ML, Li TT, Zhao YJ. Two novel mutations in the BCKDHB gene (R170H, Q346R) cause the classic form of maple syrup urine disease (MSUD). Gene. 2012 Apr 25;498(1):112–5.
420. Jaafar N, Moleirinho A, Kerkeni E, Monastiri K, Seboui H, Amorim A, et al. Molecular characterization of maple syrup urine disease patients from Tunisia. Gene. 2013 Mar 15;517(1):116–9.
421. Li X, Ding Y, Liu Y, Ma Y, Song J, Wang Q, et al. Eleven novel mutations of the BCKDHA, BCKDHB and DBT genes associated with maple syrup urine disease in the Chinese population: Report on eight cases. Eur J Med Genet. 2015 Nov;58(11):617–23.
422. Manara R, Del Rizzo M, Burlina AP, Bordugo A, Citton V, Rodriguez-Pombo P, et al. Wernicke-like encephalopathy during classic maple syrup urine disease decompensation. J Inherit Metab Dis. 2012 May;35(3):413–7.
423. Feier FH, Miura IK, Fonseca EA, Porta G, Pugliese R, Porta A, et al. Successful domino liver transplantation in maple syrup urine disease using a related living donor. Braz J Med Biol Res. 2014 Jun;47(6):522–6.
424. Guo Y, Liming L, Jiang L. Two novel compound heterozygous mutations in the BCKDHB gene that cause the intermittent form of maple syrup urine disease. Metab Brain Dis. 2015 Dec;30(6):1395–400.
425. Roilides I, Xinias I, Mavroudi A, Ioannou H, Savopoulou P, Imvrios G. Heterozygous liver transplantation for maple syrup urine disease: First European reported case. Pediatr Transplant. 2016 Sep;20(6):846–50.
426. Su L, Lu Z, Li F, Shao Y, Sheng H, Cai Y, et al. Two homozygous mutations in the exon 5 of BCKDHB gene that may cause the classic form of maple syrup urine disease. Metab Brain Dis. 2017 Jun;32(3):765–72.
427. Boros Á, Pankovics P, Kőmíves S, Liptai Z, Dobner S, Ujhelyi E, et al. Co-infection with coxsackievirus A5 and norovirus GII.4 could have been the trigger of the first episode of severe acute encephalopathy in a six-year-old child with the intermittent form of maple syrup urine disease (MSUD). Arch Virol. 2017 Jun;162(6):1757–63.
428. Herden U, Li J, Fischer L, Brinkert F, Blohm M, Santer R, et al. The first case of domino-split-liver transplantation in maple syrup urine disease. Pediatr Transplant. 2017 Sep;21(6).
429. Takano C, Ishige M, Ogawa E, Usui H, Kagawa R, Tajima G, et al. A case of classical maple syrup urine disease that was successfully managed by living donor liver transplantation. Pediatr Transplant. 2017 Aug;21(5).
430. Uaariyapanichkul J, Saengpanit P, Damrongphol P, Suphapeetiporn K, Chomtho S. Skin Lesions Associated with Nutritional Management of Maple Syrup Urine Disease. Case Rep Dermatol Med. 2017;2017:3905658.
431. Grünert SC, Rosenbaum-Fabian S, Schumann A, Schwab KO, Mingirulli N, Spiekerkoetter U. Successful pregnancy in maple syrup urine disease: a case report and review of the literature. Nutr J. 2018 May 12;17(1):51.
432. Han B, Han B, Guo B, Liu Y, Cao Z. Two novel mutations in the BCKDHB gene that cause maple syrup urine disease. Pediatr Neonatol. 2018 Oct;59(5):515–9.
433. Baştürk A, Keçeli M, Erbiş H, Soyucen E, Aliosmanoğlu İ, Dinçkan A, et al. Liver transplantation from a live donor to a patient with maple syrup urine disease: Two case reports. Turk Pediatri Ars. 2018 Jun;53(2):113–6.
434. Li W, Meng X, Wang W, Lv J, Sun Y, Lv Y, et al. Silico analysis of a novel mutation c.550delT in a Chinese patient with maple syrup urine disease. Clin Case Rep. 2018 Oct;6(10):1989–93.
435. Yang C, Linpeng S, Cao Y, Wu L. Identification of six novel mutations in five infants with suspected maple syrup urine disease based on blood and urine metabolism screening. Gene. 2019 Aug 20;710:9–16.
436. Dhawan SR, Saini AG, Vyas S, Attri SV. Teaching NeuroImages: When MRI is a clue in episodic ataxia. Neurology. 2019 Nov 26;93(22):e2074–5.
437. Nguyen TTN, Vu CD, Nguyen NL, Nguyen TTH, Nguyen NK, Nguyen HH. Identification of novel mutations in BCKDHB and DBT genes in Vietnamese patients with maple sirup urine disease. Mol Genet Genomic Med. 2020 Aug;8(8):e1337.
438. Hanna MG, Davis MB, Sweeney MG, Noursadeghi M, Ellis CJ, Elliot P, et al. Generalized chorea in two patients harboring the Friedreich’s ataxia gene trinucleotide repeat expansion. Mov Disord. 1998 Mar;13(2):339–40.
439. Forrest SM, Knight M, Delatycki MB, Paris D, Williamson R, King J, et al. The correlation of clinical phenotype in Friedreich ataxia with the site of point mutations in the FRDA gene. Neurogenetics. 1998 Aug;1(4):253–7.
440. McCormack ML, Guttmann RP, Schumann M, Farmer JM, Stolle CA, Campuzano V, et al. Frataxin point mutations in two patients with Friedreich’s ataxia and unusual clinical features. J Neurol Neurosurg Psychiatry. 2000 May;68(5):661–4.
441. Potter NT, Miller CA, Anderson IJ. Mutation detection in an equivocal case of Friedreich’s ataxia. Pediatr Neurol. 2000 May;22(5):413–5.
442. Illarioshkin SN, Bagieva GK, Klyushnikov SA, Ovchinnikov I V, Markova ED, Ivanova-Smolenskaya IA. Different phenotypes of Friedreich’s ataxia within one “pseudo-dominant” genealogy: relationships between trinucleotide (GAA) repeat lengths and clinical features. Eur J Neurol. 2000 Sep;7(5):535–40.
443. Cuda G, Mussari A, Concolino D, Costanzo FS, Strisciuglio P. Co-existence of frataxin and cardiac troponin T gene mutations in a child with Friedreich Ataxia and familial hypertrophic cardiomyopathy. Hum Mutat. 2002 Mar;19(3):309–10.
444. Zhu D, Burke C, Leslie A, Nicholson GA. Friedreich’s ataxia with chorea and myoclonus caused by a compound heterozygosity for a novel deletion and the trinucleotide GAA expansion. Mov Disord. 2002 May;17(3):585–9.
445. Gallagher CL, Waclawik AJ, Beinlich BR, Harding CO, Pauli RM, Poirer J, et al. Friedreich’s ataxia associated with mitochondrial myopathy: clinicopathologic report. J Child Neurol. 2002 Jun;17(6):453–6.
446. Hou JGG, Jankovic J. Movement disorders in Friedreich’s ataxia. J Neurol Sci. 2003 Jan 15;206(1):59–64.
447. Harris-Love MO, Siegel KL, Paul SM, Benson K. Rehabilitation management of Friedreich ataxia: lower extremity force-control variability and gait performance. Neurorehabil Neural Repair. 2004 Jun;18(2):117–24.
448. Golomb MR, Illner A, Christensen CK, Walsh LE. A child with Friedreich’s ataxia and epilepsy. J Child Neurol. 2005 Mar;20(3):248–50.
449. Gellera C, Castellotti B, Mariotti C, Mineri R, Seveso V, Didonato S, et al. Frataxin gene point mutations in Italian Friedreich ataxia patients. Neurogenetics. 2007 Nov;8(4):289–99.
450. Bernard G, Shevell M. The wobbly child: an approach to inherited ataxias. Semin Pediatr Neurol. 2008 Dec;15(4):194–208.
451. Gucev Z, Tasic V, Jancevska A, Popjordanova N, Koceva S, Kuturec M, et al. Friedreich ataxia (FA) associated with diabetes mellitus type 1 and hyperthrophic cardiomyopathy. Bosn J Basic Med Sci. 2009 May;9(2):107–10.
452. Quercia N, Somers GR, Halliday W, Kantor PF, Banwell B, Yoon G. Friedreich ataxia presenting as sudden cardiac death in childhood: clinical, genetic and pathological correlation, with implications for genetic testing and counselling. Neuromuscul Disord. 2010 May;20(5):340–2.
453. Rance G, Corben LA, Delatycki MB. Auditory pathway changes mirror overall disease progress in individuals with Friedreich ataxia. J Neurol. 2012 Dec;259(12):2746–8.
454. Verma R, Gupta M. Freidreich’s ataxia with retained reflexes: a phenotype and genotype correlation. BMJ Case Rep. 2012 Dec 14;2012.
455. Ygland E, Taroni F, Gellera C, Caldarazzo S, Duno M, Soller M, et al. Atypical Friedreich ataxia in patients with FXN p.R165P point mutation or comorbid hemochromatosis. Parkinsonism Relat Disord. 2014 Aug;20(8):919–23.
456. Dhamija R, Kirmani S. A 7-year-old girl with hypertrophic cardiomyopathy and progressive scoliosis. Semin Pediatr Neurol. 2014 Jun;21(2):67–71.
457. Caron E, Burns D, Castro D, Iannaccone ST. Atypical Presentation for Friedreich Ataxia in a Child. J Clin Neuromuscul Dis. 2015 Sep;17(1):13–7.
458. Shinnick JE, Isaacs CJ, Vivaldi S, Schadt K, Lynch DR. Friedreich Ataxia and nephrotic syndrome: a series of two patients. BMC Neurol. 2016 Jan 12;16:3.
459. Hoffman-Zacharska D, Mazurczak T, Zajkowski T, Tataj R, Górka-Skoczylas P, Połatyńska K, et al. Friedreich ataxia is not only a GAA repeats expansion disorder: implications for molecular testing and counselling. J Appl Genet. 2016 Aug;57(3):349–55.
460. Becker AB, Qian J, Gelman BB, Yang M, Bauer P, Koeppen AH. Heart and Nervous System Pathology in Compound Heterozygous Friedreich Ataxia. J Neuropathol Exp Neurol. 2017 Aug 1;76(8):665–75.
461. Barcia G, Rachid M, Magen M, Assouline Z, Koenig M, Funalot B, et al. Pitfalls in molecular diagnosis of Friedreich ataxia. Eur J Med Genet. 2018 Aug;61(8):455–8.
462. Harvey EA, Jones KS. Child Neurology: Friedreich ataxia with upper motor neuron findings: A case study. Neurology. 2018 Aug 28;91(9):426–8.
463. Clark E, Strawser C, Schadt K, Lynch DR. Identification of a novel missense mutation in Friedreich’s ataxia -FXNW 168R. Ann Clin Transl Neurol. 2019 Apr;6(4):812–6.
464. Naruse H, Takahashi Y, Ishiura H, Matsukawa T, Mitsui J, Ichikawa Y, et al. Prominent spasticity and hyperreflexia of the legs in a nepalese patient with Friedreich Ataxia. Internal Medicine. 2019;58(19).
465. Pelliccia V, Ferranti S, Mostardini R, Grosso S. A case of Friedreich ataxia in an adolescent with 16p11.2 microdeletion syndrome. Vol. 41, Neurological Sciences. 2020.
466. Hirano M, Tamaru Y, Nagai Y, Ito H, Imai T, Ueno S. Exon skipping caused by a base substitution at a splice site in the GTP cyclohydrolase I gene in a Japanese family with hereditary progressive dystonia dopa responsive dystonia. Biochem Biophys Res Commun. 1995 Aug 15;213(2):645–51.
467. Hirano M, Tamaru Y, Ito H, Matsumoto S, Imai T, Ueno S. Mutant GTP cyclohydrolase I mRNA levels contribute to dopa-responsive dystonia onset. Ann Neurol. 1996 Nov;40(5):796–8.
468. Hirano M, Imaiso Y, Ueno S. Differential splicing of the GTP cyclohydrolase I RNA in dopa-responsive dystonia. Biochem Biophys Res Commun. 1997 May 19;234(2):316–9.
469. Imaiso Y, Taniwaki T, Yamada T, Yoshimura T, Hirano M, Ueno S, et al. A novel mutation of the GTP-cyclohydrolase I gene in a patient with hereditary progressive dystonia/dopa-responsive dystonia. Neurology. 1998 Feb;50(2):517–9.
470. Tamaru Y, Hirano M, Ito H, Kawamura J, Matsumoto S, Imai T, et al. Clinical similarities of hereditary progressive/dopa responsive dystonia caused by different types of mutations in the GTP cyclohydrolase I gene. J Neurol Neurosurg Psychiatry. 1998 Apr;64(4):469–73.
471. Nitschke M, Steinberger D, Heberlein I, Otto V, Müller U, Vieregge P. Dopa responsive dystonia with Turner’s syndrome: clinical, genetic, and neuropsychological studies in a family with a new mutation in the GTP-cyclohydrolase I gene. J Neurol Neurosurg Psychiatry. 1998 Jun;64(6):806–8.
472. Furukawa Y, Kish SJ, Bebin EM, Jacobson RD, Fryburg JS, Wilson WG, et al. Dystonia with motor delay in compound heterozygotes for GTP-cyclohydrolase I gene mutations. Ann Neurol. 1998 Jul;44(1):10–6.
473. Hirano M, Yanagihara T, Ueno S. Dominant negative effect of GTP cyclohydrolase I mutations in dopa-responsive hereditary progressive dystonia. Ann Neurol. 1998 Sep;44(3):365–71.
474. Sasaki R, Naito Y, Kuzuhara S. A novel de novo point mutation in the GTP cyclohydrolase I gene in a Japanese patient with hereditary progressive and dopa responsive dystonia. J Neurol Neurosurg Psychiatry. 1998 Dec;65(6):947.
475. Hirano M, Komure O, Ueno S. A novel missense mutant inactivates GTP cyclohydrolase I in dopa-responsive dystonia. Neurosci Lett. 1999 Feb 5;260(3):181–4.
476. Brique S, Destée A, Lambert JC, Mouroux V, Delacourte A, Amouyel P, et al. A new GTP-cyclohydrolase I mutation in an unusual dopa-responsive dystonia, familial form. Neuroreport. 1999 Feb 25;10(3):487–91.
477. Weber Y, Steinberger D, Deuschl G, Benecke R, Müller U. Two previously unrecognized splicing mutations of GCH1 in Dopa-responsive dystonia: exon skipping and one base insertion. Neurogenetics. 1997 Sep;1(2):125–7.
478. Tassin J, Dürr A, Bonnet AM, Gil R, Vidailhet M, Lücking CB, et al. Levodopa-responsive dystonia. GTP cyclohydrolase I or parkin mutations? Brain. 2000 Jun;123 ( Pt 6):1112–21.
479. Ueno S, Hirano M. Missense mutants inactivate guanosine triphosphate cyclohydrolase I in hereditary progressive dystonia. Brain Dev. 2000 Sep;22 Suppl 1:S111-4.
480. Grimes DA, Barclay CL, Duff J, Furukawa Y, Lang AE. Phenocopies in a large GCH1 mutation positive family with dopa responsive dystonia: confusing the picture? J Neurol Neurosurg Psychiatry. 2002 Jun;72(6):801–4.
481. Kang JH, Kang SY, Kang HK, Koh YS, Im JH, Lee MC. A novel missense mutation of the GTP cyclohydrolase I gene in a Korean family with hereditary progressive dystonia/dopa-responsive dystonia. Brain Dev. 2004 Aug;26(5):287–91.
482. Garavaglia B, Invernizzi F, Carbone MLA, Viscardi V, Saracino F, Ghezzi D, et al. GTP-cyclohydrolase I gene mutations in patients with autosomal dominant and recessive GTP-CH1 deficiency: identification and functional characterization of four novel mutations. J Inherit Metab Dis. 2004;27(4):455–63.
483. Furukawa Y, Filiano JJ, Kish SJ. Amantadine for levodopa-induced choreic dyskinesia in compound heterozygotes for GCH1 mutations. Mov Disord. 2004 Oct;19(10):1256–8.
484. Bianca S, Bianca M. A new deletion in autosomal dominant guanosine triphosphate cyclohydrolase I deficiency gene--Segawa disease. J Neural Transm (Vienna). 2006 Feb;113(2):159–62.
485. López-Laso E, Camino R, Mateos ME, Pérez-Navero JL, Ochoa JJ, Lao-Villadóniga JI, et al. Dopa-responsive infantile hypokinetic rigid syndrome due to dominant guanosine triphosphate cyclohydrolase 1 deficiency. J Neurol Sci. 2007 May 15;256(1–2):90–3.
486. De Rosa A, Carducci C, Antonozzi I, Giovanniello T, Xhoxhi E, Criscuolo C, et al. A novel mutation in GCH-1 gene in a case of dopa-responsive dystonia. J Neurol. 2007 Aug;254(8):1133–4.
487. Cheyette BNR, Cheyette SNR, Cusmano-Ozog K, Enns GM. Dopa-responsive dystonia presenting as delayed and awkward gait. Pediatr Neurol. 2008 Apr;38(4):273–5.
488. Kim YS, Choi YB, Lee JH, Yang SH, Cho JH, Shin CH, et al. Predisposition of genetic disease by modestly decreased expression of GCH1 mutant allele. Exp Mol Med. 2008 Jun 30;40(3):271–5.
489. Ikeda T, Kanmura K, Kodama Y, Sawada K, Nunoi H, Hasegawa K. Segawa disease with a novel heterozygous mutation in exon 5 of the GCH-1 gene (E183K). Brain Dev. 2009 Feb;31(2):173–5.
490. Bodzioch M, Lapicka-Bodzioch K, Rudzinska M, Pietrzyk JJ, Bik-Multanowski M, Szczudlik A. Severe dystonic encephalopathy without hyperphenylalaninemia associated with an 18-bp deletion within the proximal GCH1 promoter. Mov Disord. 2011 Feb 1;26(2):337–40.
491. Irie S, Kanazawa N, Ryoh M, Mochizuki H, Nomura Y, Segawa M. A case of parkinsonism and dopa-induced severe dyskinesia associated with novel mutation in the GTP cyclohydrolase I gene. Parkinsonism Relat Disord. 2011 Dec;17(10):769–70.
492. Tachi N, Takahashi S, Jo M, Shinoda M. A new mutation of GCH1 in triplets family with dopa-responsive dystonia. Eur J Neurol. 2011 Sep;18(9):1191–3.
493. Lee JH, Ki CS, Kim DS, Cho JW, Park KP, Kim S. Dopa-responsive dystonia with a novel initiation codon mutation in the GCH1 gene misdiagnosed as cerebral palsy. J Korean Med Sci. 2011 Sep;26(9):1244–6.
494. Tsao CY. Guanine triphosphate-cyclohydrolase 1-deficient dopa-responsive dystonia presenting as frequent falling in 2 children. J Child Neurol. 2012 Mar;27(3):389–91.
495. Sato H, Uematsu M, Endo W, Nakayama T, Kobayashi T, Hino-Fukuyo N, et al. Early replacement therapy in a first Japanese case with autosomal recessive guanosine triphosphate cyclohydrolase I deficiency with a novel point mutation. Brain Dev. 2014 Mar;36(3):268–71.
496. Sato H, Uematsu M, Endo W, Nakayama T, Kobayashi T, Hino-Fukuyo N, et al. Early replacement therapy in a first Japanese case with autosomal recessive guanosine triphosphate cyclohydrolase I deficiency with a novel point mutation. Brain Dev. 2014 Mar;36(3):268–71.
497. Fan Z, Greenwood R, Felix ACG, Shiloh-Malawsky Y, Tennison M, Roche M, et al. GCH1 heterozygous mutation identified by whole-exome sequencing as a treatable condition in a patient presenting with progressive spastic paraplegia. J Neurol. 2014 Mar;261(3):622–4.
498. Lin Y, Wang DN, Chen WJ, Lin X, Lin MT, Wang N. Growth hormone deficiency in a dopa-responsive dystonia patient with a novel mutation of guanosine triphosphate cyclohydrolase 1 gene. J Child Neurol. 2015 May;30(6):796–9.
499. Kim JI, Choi JK, Lee JW, Kim J, Ki CS, Hong JY. A novel missense mutation in GCH1 gene in a Korean family with Segawa disease. Brain Dev. 2015 Mar;37(3):359–61.
500. Sun Z fang, Zhang Y han, Guo J feng, Sun Q ying, Mei J pu, Zhou H lin, et al. Genetic diagnosis of two dopa-responsive dystonia families by exome sequencing. PLoS One. 2014;9(9):e106388.
501. Zhang W, Zhou Z, Li X, Huang Y, Li T, Lin Y, et al. Dopa-responsive dystonia in Chinese patients: Including a novel heterozygous mutation in the GCH1 gene with an intermediate phenotype and one case of prenatal diagnosis. Neurosci Lett. 2017 Mar 22;644:48–54.
502. Yang CC, Wang WC, Yeh TH, Chen TH, Liu YL, Lu MK, et al. A novel missense mutation of the GTP cyclohydrolase 1 gene in a Taiwanese family with dopa-responsive dystonia: A case report. Clin Neurol Neurosurg. 2018 Feb;165:21–3.
503. Wu-Chou YH, Yeh TH, Wang CY, Lin JJ, Huang CC, Chang HC, et al. High frequency of multiexonic deletion of the GCH1 gene in a Taiwanese cohort of dopa-response dystonia. Am J Med Genet B Neuropsychiatr Genet. 2010 Jun 5;153B(4):903–8.
504. Gowda VK, Nagarajan B, Srinivasan VM, Benakappa A. A Novel GCH1 Mutation in An Indian Child with GTP Cyclohydrolase Deficiency. Indian J Pediatr. 2019 Aug;86(8):752–3.
505. Flotats-Bastardas M, Hebert E, Raspall-Chaure M, Munell F, Macaya A, Lohmann K. Novel GCH1 Compound Heterozygosity Mutation in Infancy-Onset Generalized Dystonia. Neuropediatrics. 2018 Aug;49(4):296–7.
506. Giri S, Naiya T, Roy S, Das G, Wali GM, Das SK, et al. A Compound Heterozygote for GCH1 Mutation Represents a Case of Atypical Dopa-Responsive Dystonia. J Mol Neurosci. 2019 Jun;68(2):214–20.
507. Eye PG, Horvat D, Wade KT, Hack NK, Dennison DH. Pure hypotonia in a four-year-old patient: An atypical presentation of Dopa-responsive dystonia. J Neurol Sci. 2019 Aug 15;403:125–6.
508. Wang X, Mei S, Tian Z, Wang L, Hao G, Zhu X, et al. Case Report: Clinical Outcome From Pallidal Stimulation in a Patient With Levodopa-Resistant Dopa-Responsive Dystonia. Front Neurol. 2022;13:921577.
509. Chen Y, Liu K, Yang Z, Wang Y, Zhou H. Case Report: Severe Hypotonia Without Hyperphenylalaninemia Caused by a Homozygous GCH1 Variant: A Case Report and Literature Review. Front Genet. 2022;13:929069.
510. Scola RH, Carducci C, Amaral VG, Lorenzoni PJ, Teive HAG, Giovanniello T, et al. A novel missense mutation pattern of the GCH1 gene in dopa-responsive dystonia. Arq Neuropsiquiatr. 2007 Dec;65(4B):1224–7.
511. Teva Galán MD, Esteban Cantó V, Picó Alfonso N, Jover Cerdá J. [GTP cyclohydrolase 1-deficient dopa-responsive hereditary dystonia]. An Pediatr (Barc). 2011 Jul;75(1):55–7.
512. López-Laso E, Beyer K, Opladen T, Artuch R, Saunders-Pullman R. Dyskinesias as a limiting factor in the treatment of Segawa disease. Pediatr Neurol. 2012 Jun;46(6):404–6.
513. Jain R, Shukla B, Mittal M. Delayed Diagnosis of Dopa responsive Dystonia in Two Siblings. Indian Pediatr. 2016 May 8;53(5):427–8.
514. Yang CC, Wang WC, Yeh TH, Chen TH, Liu YL, Lu MK, et al. A novel missense mutation of the GTP cyclohydrolase 1 gene in a Taiwanese family with dopa-responsive dystonia: A case report. Clin Neurol Neurosurg. 2018 Feb;165:21–3.
515. Bendi VS, Shou J, Joy S, Torres-Russotto D. Motor fluctuations and levodopa-induced dyskinesias in dopa-responsive dystonia. Parkinsonism Relat Disord. 2018 May;50:126–7.
516. Edvardson S, Shaag A, Kolesnikova O, Gomori JM, Tarassov I, Einbinder T, et al. Deleterious mutation in the mitochondrial arginyl-transfer RNA synthetase gene is associated with pontocerebellar hypoplasia. Am J Hum Genet. 2007 Oct;81(4):857–62.
517. Rankin J, Brown R, Dobyns WB, Harington J, Patel J, Quinn M, et al. Pontocerebellar hypoplasia type 6: A British case with PEHO-like features. Am J Med Genet A. 2010 Aug;152A(8):2079–84.
518. Glamuzina E, Brown R, Hogarth K, Saunders D, Russell-Eggitt I, Pitt M, et al. Further delineation of pontocerebellar hypoplasia type 6 due to mutations in the gene encoding mitochondrial arginyl-tRNA synthetase, RARS2. J Inherit Metab Dis. 2012 May;35(3):459–67.
519. Kastrissianakis K, Anand G, Quaghebeur G, Price S, Prabhakar P, Marinova J, et al. Subdural effusions and lack of early pontocerebellar hypoplasia in siblings with RARS2 mutations. Arch Dis Child. 2013 Dec;98(12):1004–7.
520. Joseph JT, Innes AM, Smith AC, Vanstone MR, Schwartzentruber JA, Bulman DE, et al. Neuropathologic features of pontocerebellar hypoplasia type 6. J Neuropathol Exp Neurol. 2014 Nov;73(11):1009–25.
521. Li Z, Schonberg R, Guidugli L, Johnson AK, Arnovitz S, Yang S, et al. A novel mutation in the promoter of RARS2 causes pontocerebellar hypoplasia in two siblings. J Hum Genet. 2015 Jul;60(7):363–9.
522. Lax NZ, Alston CL, Schon K, Park SM, Krishnakumar D, He L, et al. Neuropathologic Characterization of Pontocerebellar Hypoplasia Type 6 Associated With Cardiomyopathy and Hydrops Fetalis and Severe Multisystem Respiratory Chain Deficiency due to Novel RARS2 Mutations. J Neuropathol Exp Neurol. 2015 Jul;74(7):688–703.
523. Nishri D, Goldberg-Stern H, Noyman I, Blumkin L, Kivity S, Saitsu H, et al. RARS2 mutations cause early onset epileptic encephalopathy without ponto-cerebellar hypoplasia. Eur J Paediatr Neurol. 2016 May;20(3):412–7.
524. Ngoh A, Bras J, Guerreiro R, Meyer E, McTague A, Dawson E, et al. RARS2 mutations in a sibship with infantile spasms. Epilepsia. 2016 May;57(5):e97–102.
525. González-Serrano LE, Karim L, Pierre F, Schwenzer H, Rötig A, Munnich A, et al. Three human aminoacyl-tRNA synthetases have distinct sub-mitochondrial localizations that are unaffected by disease-associated mutations. J Biol Chem. 2018 Aug 31;293(35):13604–15.
526. Nevanlinna V, Konovalova S, Ceulemans B, Muona M, Laari A, Hilander T, et al. A patient with pontocerebellar hypoplasia type 6: Novel RARS2 mutations, comparison to previously published patients and clinical distinction from PEHO syndrome. Eur J Med Genet. 2020 Mar;63(3):103766.
527. Al Balushi A, Matviychuk D, Jobling R, Salomons GS, Blaser S, Mercimek-Andrews S. Phenotypes and genotypes of mitochondrial aminoacyl-tRNA synthetase deficiencies from a single neurometabolic clinic. JIMD Rep. 2020 Jan;51(1):3–10.
528. Jiang HF, Deng J, Fang F, Li H, Wang XH, Dai LF. [Early onset epileptic encephalopathy caused by mitochondrial arginyl-tRNA synthetase gene deficiency: report of two cases and literature review]. Zhonghua Er Ke Za Zhi. 2020 Nov 2;58(11):893–9.
529. Xu Y, Wu BB, Wang HJ, Zhou SZ, Cheng GQ, Zhou YF. A term neonate with early myoclonic encephalopathy caused by RARS2 gene variants: a case report. Transl Pediatr. 2020 Oct;9(5):707–12.
530. de Valles-Ibáñez G, Hildebrand MS, Bahlo M, King C, Coleman M, Green TE, et al. Infantile-onset myoclonic developmental and epileptic encephalopathy: A new RARS2 phenotype. Epilepsia Open. 2022 Mar;7(1):170–80.
531. Sevinç S, İnci A, Ezgü FS, Eminoğlu FT. A Patient with a Novel RARS2 Variant Exhibiting Liver Involvement as a New Clinical Feature and Review of the Literature. Mol Syndromol. 2022 May;13(3):226–34.
532. Alicea Marrero MM, Español M, Marrero-Rivera G, Bauchat A, Cruz V, Yu L, et al. Successful Haploidentical Bone Marrow Transplantation of an Infant With a Novel Mutation in SAMD9L Gene (Ataxia-Pancytopenia Syndrome). J Pediatr Hematol Oncol. 2022 Oct 1;44(7):419–20.
533. Thunström S, Axelsson M. Leukoencephalopathia, demyelinating peripheral neuropathy and dural ectasia explained by a not formerly described de novo mutation in the SAMD9L gene, ends 27 years of investigations - a case report. BMC Neurol. 2019 May 3;19(1):89.
534. Bowden R, Davies RW, Heger A, Pagnamenta AT, de Cesare M, Oikkonen LE, et al. Sequencing of human genomes with nanopore technology. Nat Commun. 2019 Apr 23;10(1):1869.
535. Cheah JJC, Brown AL, Schreiber AW, Feng J, Babic M, Moore S, et al. A novel germline SAMD9L mutation in a family with ataxia-pancytopenia syndrome and pediatric acute lymphoblastic leukemia. Haematologica. 2019 Jul;104(7):e318–21.
